# Colloidal adsorption in planar polymeric brushes†

**DOI:** 10.1039/d3na00598d

**Published:** 2023-10-23

**Authors:** Clemens Franz Vorsmann, Sara Del Galdo, Barbara Capone, Emanuele Locatelli

**Affiliations:** a Dipartimento di Fisica e Astronomia, Università di Padova Sezione di Padova, via Marzolo 8 I-35131 Padova 2INFN Italy emanuele.locatelli@unipd.it; b INFN Sezione di Padova, via Marzolo 8 I-35131 Padova Italy; c Science Department, University of Roma Tre Via della Vasca Navale 84 00146 Rome Italy

## Abstract

The design of nano-functionalised membranes or channels, able to effectively adsorb pollutants in aqueous solutions, is a topic that is gaining a great deal of attention in the materials science community. With this work we explore, through a combination of scaling theories and molecular dynamics simulations, the adsorption of spherical non-deformable colloidal nanoparticles within planar polymeric brushes. Our strategy is twofold: first, we generalise the Alexander-de Gennes theory for planar homopolymeric brushes to the case of diblock copolymer brushes, then we map the adsorbing homopolymeric brushes onto a diblock copolymer system, where the adsorbed colloids and all interacting monomers are considered monomers in bad solvent and we apply the generalised scaling theory to this effective diblock copolymer. This allows the prediction of the average conformation of the grafted substrate, *i.e.* its average height, as a function of the amount of loaded particles, as well as the introduction of a continuous mapping between a homopolymeric brush, the fraction of loaded particles and the average height of the adsorbing substrate.

## Abstract 2

Polymer brushes interact with colloids and adsorb them. Replacing colloids with monomers in poor solvent, we develop an analytical theory for diblock copolymer brushes and remap the system to a blob model that allows for scaling predictions.

## Graphical abstract

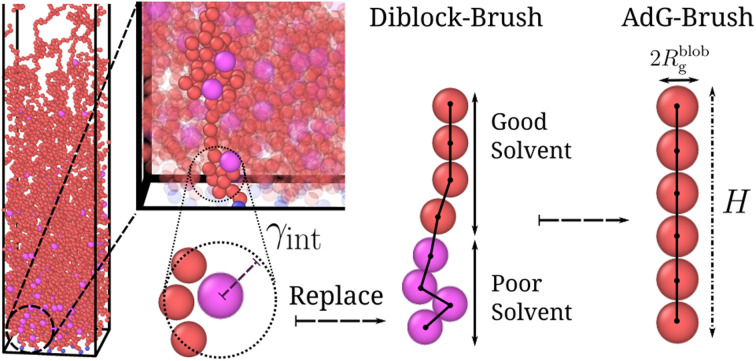

## 1 Introduction

Polymer brushes are assemblies of polymer chains, tethered by one end to a solid surface *via* either chemical bonding or physical adsorption to the surface;^1,2^ the resulting polymer films are typically tens to hundreds of nanometres thick. These polymer-based monolayers can be integrated into devices for nano-technological applications: advances in the development of controlled polymerisation processes, combined with the refinement of supramolecular strategies, allow a high degree of control over the composition, architecture and length of the anchored macromolecular chains, as well as the grafting density.^1,3^ Polymer brushes have hence attracted the attention of the materials science community for the wide range of their possible applications which include sensing, drug delivery, anti-fouling coatings and lubrication.^4^ Moreover, coatings of polymer brushes can also be exploited to impart stimuli-responsive properties to surfaces, depending on the system used for functionalisation.^5,6^

The most common application of polymer brushes today is the functionalisation of membranes (and/or channels) for separation and purification processes.^4,7^ Examples include protein separation/purification (*i.e.* affinity membrane chromatography),^8^ dehydration of organic solvents (*i.e.* pervaporation),^9^ gas separation^10^ and water remediation.

Indeed, membrane adsorption has become one of the preferred methods for removing toxic contaminants from wastewater. Alternative methods include nanofiltration and ultrafiltration, reverse osmosis, chemical precipitation, electrochemical treatments and extraction. These techniques are often more expensive – as they typically require large quantities of reagents – and less effective than the use of adsorptive membranes. In fact, membranes are typically highly permeable to water fluxes, do not require high pressures to operate, can be regenerated and take up little space. In addition, the use of membranes does not suffer from the problem of recovery of solvent or surfactants or polymers in solution.^11,12^

From a microscopic point of view, membranes are characterised by a large adsorption area (implying a large number of adsorption sites per unit area) and they can be specifically designed and functionalised to optimise their efficiency, cost and versatility. However, a critical issue for the use of these nano-functionalised membranes is the optimisation of the amount of adsorbent species that can be incorporated into the membrane, either as the number of adsorption sites per polymer chain or as the number of anchored chains. In fact, the density of the grafted macromolecules should be optimised to maximise the number of adsorption sites while avoiding agglomeration and clogging of the membranes.

From a theoretical as well as experimental perspective, considerable interest has been devoted to the limiting case in which there is no chemical affinity between monomers and nano-particles. In such a scenario, adsorption is possible *via* entropic mechanisms.^13^ However, for most applications it is desirable to also optimise the enthalpic interactions between the polymer and the adsorbate; coupling it with other properties, *e.g.* thermo-sensitivity, endows the brush with stimuli-responsive mechanisms that can be exploited in many ways. Theoretically, a mean-field free energy description of such a system has been proposed;^14–16^ however, due to its mean-field nature, it compares quantitatively against simulation results only in the limit of weak attraction.

We present here a theoretical and computational study of the adsorption of waste particles or small ligands in adsorptive membranes in solution. Using a combination of scaling theories and molecular dynamics simulations (MD), we study the adsorption of spherical colloidal nanoparticles (mimicking waste particles/ligands) within model polymer brushes by varying the strength of the interaction between the polymer and colloids and the grafting density. The central core of this work is the definition of a novel theoretical framework that allows the derivation of a simplified description of the process of adsorption of colloidal nanoparticles within planar homopolymeric brushes. In particular, the scope of this work is to find simple measurable macroscopic changes that can be used as an insight to quantify adsorption. Starting from the known results for homopolymeric brushes in good solvent, we introduce the adsorption process as a sort of perturbation to the system; when colloids are adsorbed within a polymeric brush, the nanoparticles interact with a selection of monomers belonging to the grafted macromolecules. The colloids act as an effective glue between the monomers, whose collapse around the adsorbed particle can be foreseen as a local change (worsening) in solvent quality. The paper is structured as follows. In Section 2, we report the description of the numerical model and the simulation details; then, in Section 3, we present the generalisation of the Alexander-de Gennes scaling theory to diblock copolymer brushes. After validating our reference simulations in Section 4.1, we discuss the results of the simulations and what we can learn from them in Section 4.2. Using this insight, we successfully compare theoretical prediction with numerical simulations in Section 4.3, detailing the mapping between the monomer-resolved system and the homopolymeric effective representation that allows the correct scaling. Finally, we show that the Alexander-de Gennes scaling relation remains valid, if the proper scaling factor (*i.e.* the gyration radius of an equivalent diblock copolymer) is considered.

## 2 Materials and methods

We consider a polymeric brush, composed of linear chains grafted onto a perfectly flat impenetrable surface; we also consider colloids, acting as “adsorbates”, diffusing in the system. We set the thermal energy *k*_B_*T* = 1/*β* = 1 as the unit of energy. All monomers and colloids interact *via* the Lennard–Jones (LJ) interaction potential1
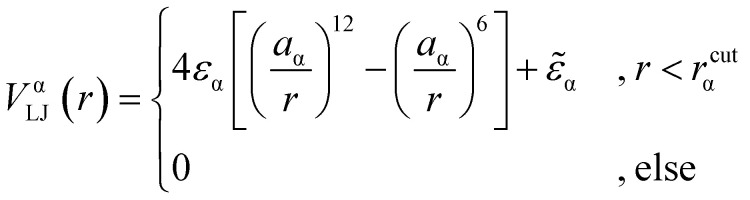


Indicating with the subscript c the colloids, m the monomers and cm the mixed case, the LJ-sizes are *a*_m_ = *a* = 1.0, and is thus set as the unit of length, *a*_c_ = 1.5*a* and 
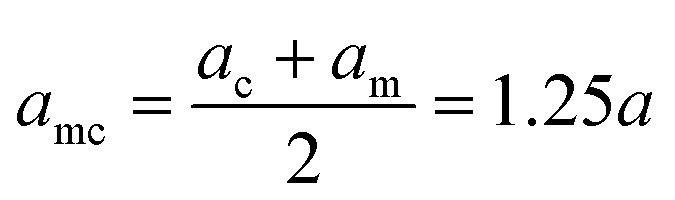
. We choose a colloid size that is only slightly larger than the monomer size. Such a size ratio has been shown to reproduce, at least qualitatively within a model system, adsorption of heavy metal ions, whose removal is of capital importance in water remediation, by polymer functionalised nanoparticles.^17^ The parameter *ε̃*_α_ is chosen such that the value of the potential energy is zero at *r*^cut^_α_, ensuring a smooth transition of the piece-wise defined potential without affecting the force. We consider a polymer brush in good solvent, whose monomers act as adsorption sites for the suspended colloidal particles. Thus, monomer–monomer as well as colloid–colloid interactions are chosen as purely repulsive *r*^cut^_c_ = 2^1/6^*a*_c_ and *r*^cut^_m_ = 2^1/6^*a*_m_; instead, we introduce attraction in the mixed case by setting *r*^cut^_mc_ = 2*a*_mc_. The depth of the LJ potential is *ε*_c_ = *ε*_m_ = 1.0*k*_B_*T* while, for *ε*_mc_, we explore a range of values *ε*_mc_ ∈ {1.0, 1.5, 2.0, 4.0}*k*_B_*T* in order to assess the role of the monomer–colloid interaction energy. Neighbouring monomers along the grafted polymer chains are held together by means of the finite extensible nonlinear elastic (FENE) potential:^18^2
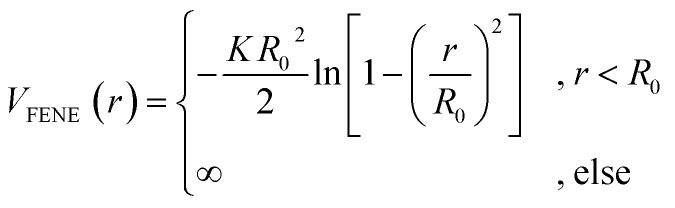
where *K* = 30*ε*_m_/*a*^2^ and *R*_0_ = 1.5*a*. We simulate a system in a box of the size *V* = *L*_*x*_*L*_*y*_*L*_*z*_, with *L*_*x*_ = *L*_*y*_ = 20*a* and *L*_*z*_ = 200*a*, with periodic boundary conditions in the *x*- and *y*-directions; the polymer chains are grafted at *z* = 0 and effectively occupy a fraction of the available space. The perfectly flat, impenetrable plane at *z* = 0 is implemented using again a LJ potential, with *ε*_w_ = *ε*_m_ and *a*_w_ = *a*, acting on all monomers (except, for simplicity, the grafted ones) and all colloids. We construct the system by first placing the grafting points of the polymers in a random manner using Poisson disk sampling^19^ in order to obtain a spatially homogeneous grafting distribution in the *x*–*y* plane. We then grow the chain from the grafting points using a self-avoiding and boundary-avoiding random walk, in order to grow the chain away from the bottom wall. We further place *N*_c_ colloids in the upper half of the box again using Poisson disk sampling.

We perform full monomer molecular dynamics (MD) simulations using LAMMPS.^20^ Alongside the units of length and energy, we set *m* = 1 for both monomers and colloids, for simplicity; this also sets the MD time unit *τ* = 1. We integrate the equations of motion using the velocity Verlet algorithm, with integration time step Δ*t* = 0.001*τ*; simulations are performed in the NVT-ensemble employing the Langevin thermostat, with friction coefficient *γ* = 1*τ*^−1^. We neglect hydrodynamic interactions, as they are not affecting the static and thermodynamic properties in equilibrium. We simulate polymer brushes of *M* = 200 monomers at different grafting densities *σ*_g_*a*^2^ = 0.032, 0.048, 0.064, 0.08. Furthermore, we span a wide range of values for the colloid packing fraction, defined as *η*_c_ = *N*_c_*V*_c_/(*L*_*x*_*L*_*y*_*H*_0_), *V*_c_ = π*a*_c_^3^/6 being the volume occupied by a colloid and *H*_0_ being the average height of the unperturbed brush at a given *σ*_g_. We consider a colloid “adsorbed” if there is at least one monomer below a certain cut-off distance, that will be introduced later. To compare our simulation results with the theoretical predictions, we introduce the packing fraction of the adsorbed colloids *η*^a^_c_ = *N*^a^_c_*V*_c_/(*L*_*x*_*L*_*y*_*H*_0_), where *N*^a^_c_ is the number of adsorbed colloids. We verified that, for the chosen chain length and grafting densities, the brushes are always in the scaling regime (see Section 4.1). For each set of parameters we perform 5 independent realisations: for each one, from the initial configuration, we equilibrate the system until the average height of the adsorbed colloids is constant. Afterwards, we perform a production run of 800 000*τ* (8 × 10^8^ time steps).

## 3 Generalising the Alexander-de Gennes theory to adsorbing brushes

Properties such as average height, brush profile or even microphase separations, are known to be well described by means of scaling theories.^21–24^ Within this approach, it is possible to link the average properties of the brush to a combination of the single molecule radius of gyration

3 *R*_g_ ∼ *ξ*_g_*M*^*ν*^,

and the adimensional quantity *σ*_g_/σ*, where σ* = 1/*R*_g_^2^ is the grafting density at which two polymers represented through their radius of gyration would begin to overlap, *ξ*_g_ is a model dependent prefactor and *ν* is the Flory exponent. In particular by introducing the Alexander-de Gennes notation,^21,22^ the average height of the brush can be expressed as:^25^4
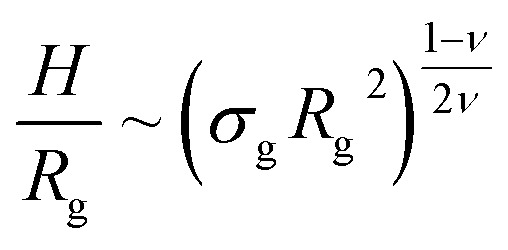


In order to generalise the AdG scaling theory to account for adsorption, we first remap a single polymer chain, that has adsorbed one or more colloids, onto a diblock copolymer; we will then use such a generalisation to rescale the height profiles of the brushes for all grafting densities after adsorption.

As mentioned, we assume that the monomers act as adsorption sites for the colloids; this, from a physical perspective, can be recast into an attractive interaction between the two species. Adsorption thus induces a local change in solvent quality as, effectively, monomers crowd around the colloidal particle and expel the solvent. This implies that the effective size of the grafted chain, upon adsorption, should account for the adsorbed colloid.

In the spirit of the AdG formalism, we aim at linking the properties of the brush to the single chain properties. Given a brush of *N* chains and *N*^a^_c_ adsorbed colloids, we name *n*_c_ = *N*_c_/*N* and *n*^a^_c_ = *N*^a^_c_/*N* the number of colloids and adsorbed colloids per chain, respectively. Upon adsorbing a colloid, a grafted homopolymeric macromolecule, initially of molecular weight *M*, can be mapped onto an “equivalent” diblock copolymer chain, made of *M*_A_ monomers in good solvent, and *M*_B_ monomers in bad solvent. The latter quantity has two contributions. The first comes from *M*_int_ = *M* − *M*_A_ monomers, interacting with the colloids. In addition, a single nanoparticle can be mapped onto a homopolymeric chain in bad solvent whose radius of gyration satisfies the relation 
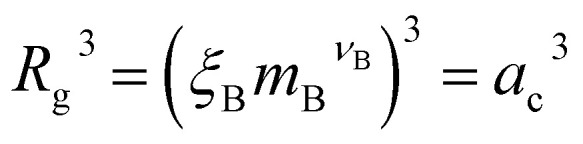
 where *a*_c_ is the radius of the colloid and *m*_B_ is the number of equivalent monomers in bad solvent.^17^ In other words, for each chain, every adsorbed colloid can be treated as an additional contribution to the total number of monomers in bad solvent. Thus, as adsorption takes place, each original homopolymeric chain made by *M* monomers is mapped onto a diblock copolymer made by *M*_A_ monomers in good solvent and *M*_B_ monomers in bad solvent

5 *M*_B_ = *M*_int_ + *n*^a^_c_*m*_B_

It is important to stress that in all considerations made, the total number of effective monomers constituting the diblock *M*^db^ = *M*_A_ + *M*_int_ + *n*^a^_c_*m*_B_ grows upon increasing the amount of adsorbed colloids. This ensures that the brush will be in the scaling regime at any point of the adsorption process. To generalise the results obtained using AdG, each A–B diblock copolymer is mapped onto an “equivalent homopolymer” *i.e.* a homopolymer whose radius of gyration is equal to that of one of the diblock copolymers.

The AdG theory requires two parameters: the grafting density and the radius of gyration of the grafted chains. We start by analysing the radius of gyration of the diblock copolymer, by approximating it as:6
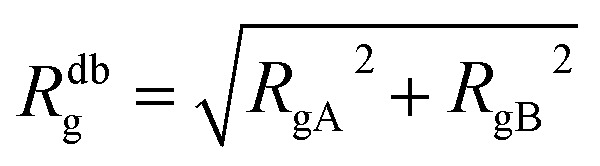
where *R*_gA_ and *R*_gB_ are given by7
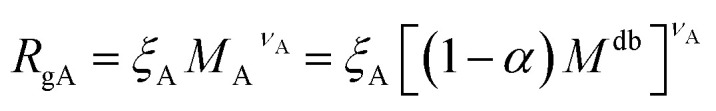

8 *R*_gB_ = *ξ*_B_[*αM*^db^]^*ν*_B_^

9
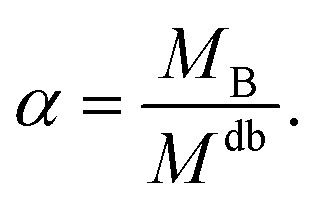


Eqn (6) can therefore be rewritten as:10
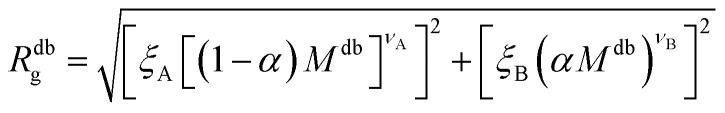


It is now possible to determine the “corresponding homopolymer”: imposing that its gyration radius is identical to that of one of the diblock copolymers *R*^eq^_g_ = *R*^db^_g_, we set the number *M*_eq_ of equivalent monomers. In this way, the reduced grafting density *σ*_g_/σ* remains the same in the two representations.

For convenience, the equivalent homopolymer will be considered to be made of monomers in good solvent, *i.e.* monomers that follow the same scaling laws as the A part of the diblock copolymer chain. We can therefore rewrite the total radius of gyration of the equivalent homopolymer as11
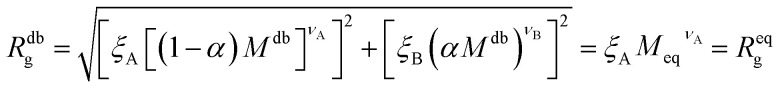
Hence12
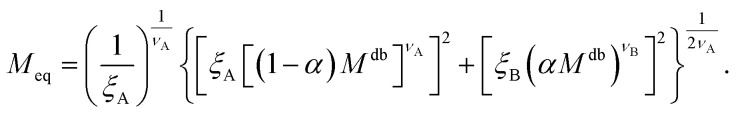


Within the AdG theory, a homopolymeric brush of grafting density *σ*_g_ can be seen as a chain of non-overlapping blobs of radius 
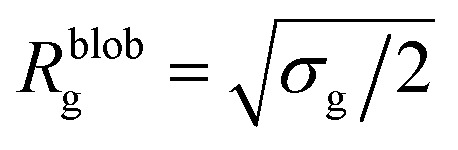
.^21,22^ Each blob contains a number of monomers 
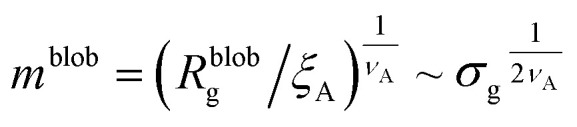
. According to our representation, the total number of blobs that will constitute the equivalent brush is given by the ratio between the equivalent length *M*_eq_ and the number of monomers per blob:

13 *M*_blob_ = *M*_eq_/*m*^blob^.

The height of the brush can be approximated as the product of the number of blobs and the diameter of the blobs:14
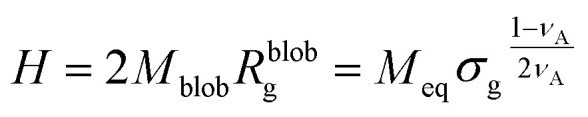
or, equivalently:15
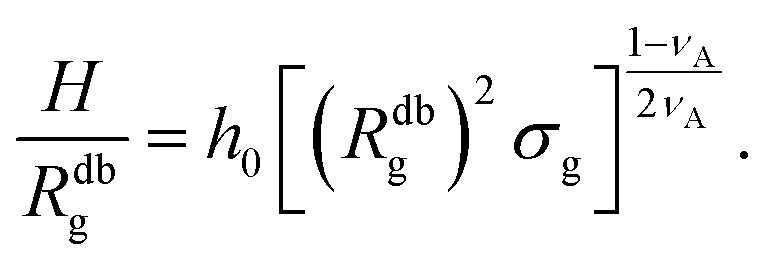
where *h*_0_ = 1.4 is obtained by fitting the data, reported in Fig. 2.

**Fig. 1:**
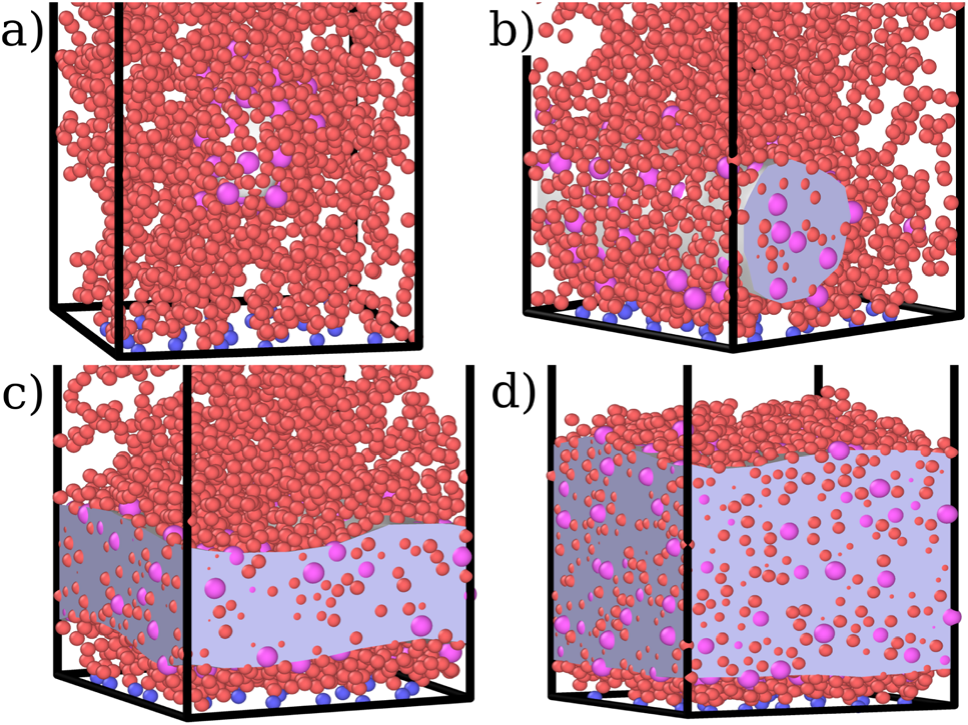
Snapshots of the simulated systems at fixed *ε*_mc_ = 4.0*k*_B_*T*, *σ*_g_*a*^2^ = 0.064 and (a) *η*_c_ = 0.002, (b)*η*_c_ = 0.006, (c) *η*_c_ = 0.013, and (d) *η*_c_ = 0.027. The polymer beads that act as grafting points are indicated in blue, monomers in red and colloids in magenta. The grey volume indicates the space occupied by the colloids; it is obtained with the open-source software OVITO as the minimal surface encompassing all the colloids.

**Fig. 2:**
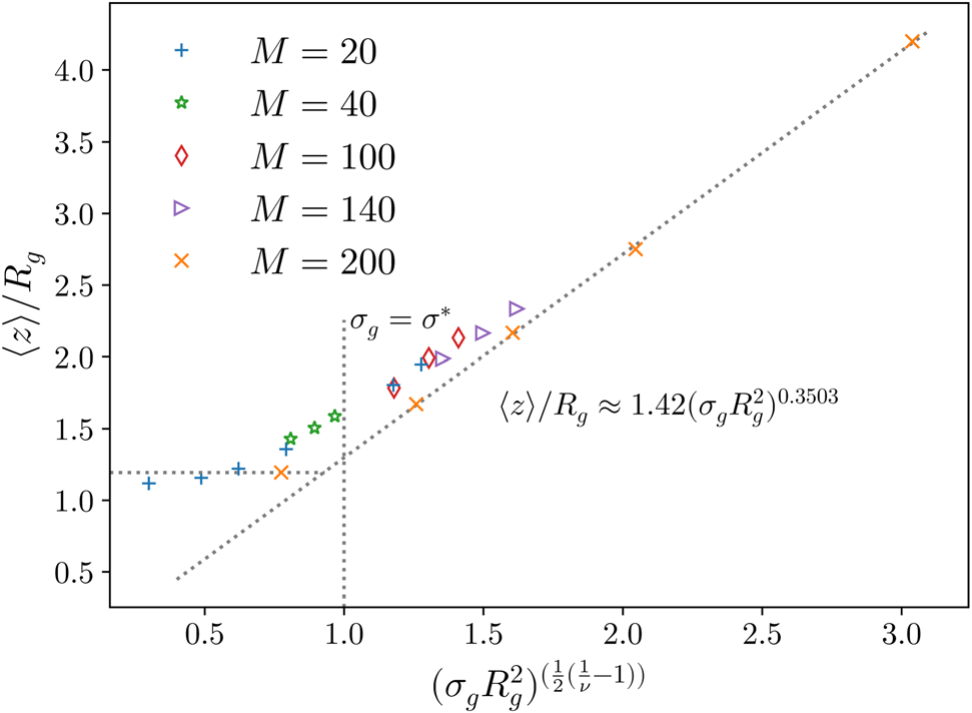
Height of the brush over the bulk gyration radius of a single chain as a function of the scaling parameter (*σ*_g_*R*_g_^2^)^(1−*ν*)/(2*ν*)^ for brushes of different lengths *M* and different grafting densities, as in ref. 25. The dashed blue line indicates the beginning of the stretched regime at 
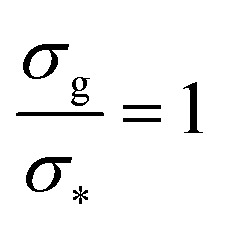
. The dotted gray lines are guides to the eye and indicate the two regimes, mushroom and stretched, predicted using the theory. Fitting the data returns the numerical prefactor *h*_0_ = 1.4.

The key point to apply eqn (15) is to know *M*_A_ and *M*_B_ as a function of the density of adsorbed colloids (per chain). We will propose, in Section 4.3, a way to estimate them, based on the phenomenology observed from the numerical simulations.

## 4 Results

### 4.1 Calibrating the reference system *via* the Alexander-de Gennes scaling

In order to compare the prediction of the scaling theory, generalised to the case of adsorption, with results from numerical simulations, one needs to study, for all values of *σ*_g_ considered, polymeric brushes well in the scaling regime.

We report, in Fig. 2, the average height of the brush, normalised by the gyration radius of a single chain, as a function of the scaled grafting density (*σ*_g_*R*_g_^2^)^(1−*ν*)/(2*ν*)^. The average height 〈*z*〉 is obtained as the average *z*-component of the chain monomer positions.^24,26,27^ Comparing Fig. 2 with results reported in the literature^25^ we find the correct scaling. We thus choose to take as a reference system a brush with *M* = 200 monomers per chain, which fits the scaling regime for a reasonable range of *σ*_g_ values. In particular, the range considered in this paper is 0.032 ≤ *σ*_g_*a*^2^ ≤ 0.08 corresponding to 1.60 ≤ (*σ*_g_*R*_g_^2^)^(1−*ν*)/(2*ν*)^ ≤ 2.21.

We further verify that the shape of the brush density profile is parabolic, as predicted by self-consistent field theory (see the ESI†). These results allow us to move further towards the generalisation of the AdG theory to the case of an adsorbing brush, with the certainty that all the corresponding homopolymeric systems reproduce the expected scaling predictions.

### 4.2 Phenomenology of colloidal adsorption from numerical simulations

In this section, we discuss the emerging phenomenology of brush adsorption, examining the results of the numerical simulations. As already mentioned, we performed an extensive investigation, by varying the monomer–colloid interaction strength *ε*_mc_ ∈ [1.0, 4.0] for the fixed grafting density *σ*_g_*a*^2^ = 0.064 as well as by varying *σ*_g_ for the fixed value *ε*_mc_ = 1.5*k*_B_*T*. We highlight that all the observables shown in this section are measured at equilibrium. As mentioned in Section 2, a colloid is considered to be adsorbed if it is close enough to at least one monomer; *vice versa*, a monomer is defined as an adsorption site if it is interacting with at least one colloid. Equilibrium is reached when the average height of the adsorbed colloids displays a stationary value in time.

**Fig. 3:**
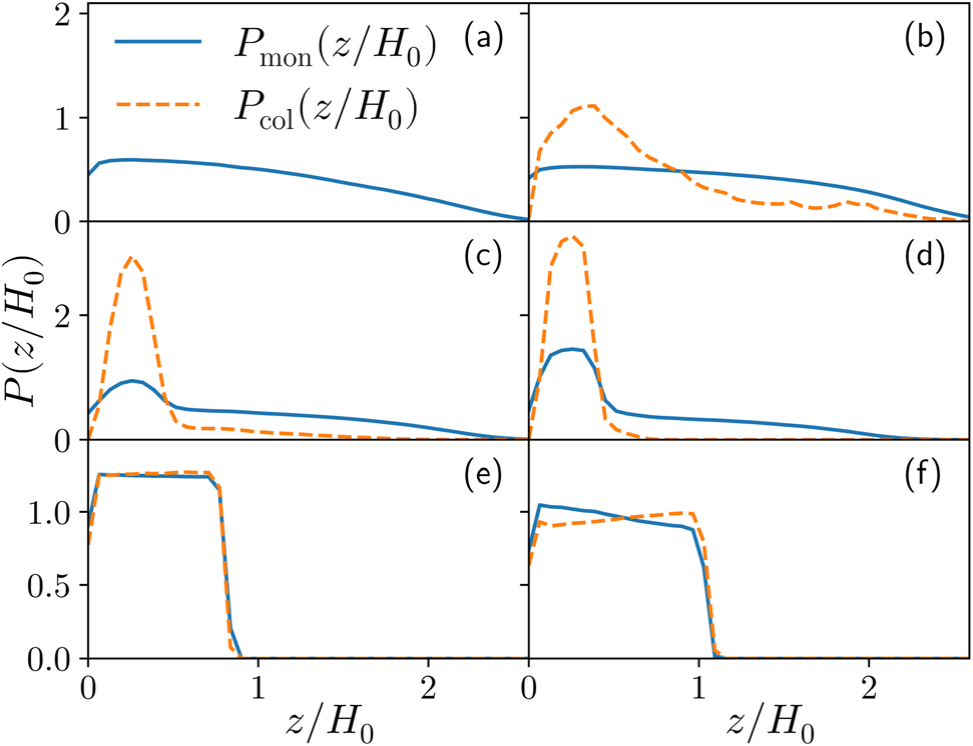
The probability density *P*(*z*) to find either a colloid (orange dashed line) or a monomer (blue full line) at height *z* as a function of the reduced height *z*/*H*_0_, *H*_0_ being height at *η*_c_ = 0, for the reference system *ε*_mc_ = 4*k*_B_*T*, and *σ*_g_*a*^2^ = 0.064 at different values of the colloid packing fraction (a) *η*_c_ = 0 (b) *η*_c_ = 0.0003 (corresponding to, for this specific system, a single colloid) (c) *η*_c_ = 0.02, (d) *η*_c_ = 0.04, (e) *η*_c_ = 0.19 and (f) *η*_c_ = 0.32.

For the sake of clarity, we chose to report one case (*σ*_g_*a*^2^ = 0.064, *ε*_mc_ = 4*k*_B_*T*) in the main article and refer to the ESI† for all the other analysed cases. In fact, although details can remain system dependent, the general features are common. Specifically, *ε*_mc_ = 4*k*_B_*T* is the interaction strength that presents more prominent adsorption and, as mentioned, *σ*_g_*a*^2^ = 0.064 reproduces the parabolic regime for the *M* = 200 case prior to adsorption, as shown in Fig. 3a.^21,22,26,27^

For both species, we measure the probability of finding either a monomer or a colloid at a certain height *z* with respect to the grafting plane. In the reported case (see Fig. 3b), when a single colloid is inserted in the simulation box (*η*_c_ = 0.0003), the adsorption probability is one, but the average shape of the brush remains almost unperturbed. Upon adsorption of a sufficient amount of colloids, the profile of the brush changes and develops a maximum in the region close to the bottom plane. At the same height we notice the concurrent accumulation of the colloids (Fig. 3c).

This co-localisation highlights the formation of a condensed phase, a droplet, made of a mixture of collapsed monomers and colloids; it is visible from the snapshots reported in Fig. 1. The droplet grows upon increasing the number of adsorbed colloids and, eventually, percolates through the periodic boundaries. At first, the brush maintains a top layer totally exposed to the solvent (that is, in good solvent), as shown in Fig. 1c and 3d; however, upon increasing adsorption, the brush collapses completely and the colloidal particles become uniformly distributed inside the (collapsed) brush (Fig. 1d and 3e). Intriguingly, in the minimal coarse-grained model here considered, even when all monomers become adsorbing sites, the brush still shows the capability of rearranging the monomers around the colloidal nano-particles and allows for further adsorption, within a range of interaction strengths. Such an increase in the number of adsorbed colloids causes the formation of a layer of colloids that deposit on the exposed surface of the collapsed brush, as shown in Fig. 3f; the average height of the loaded brush is thus increased in the process. Interestingly, the same phenomenology has been observed with another theoretical model^14^ and has also been hypothesized to explain the experimental results, obtained *via* quartz crystal microbalance measurements.^28^ Furthermore, the results obtained at small values of *ε*_mc_ are in agreement with previous simulations.^29^

**Fig. 4:**
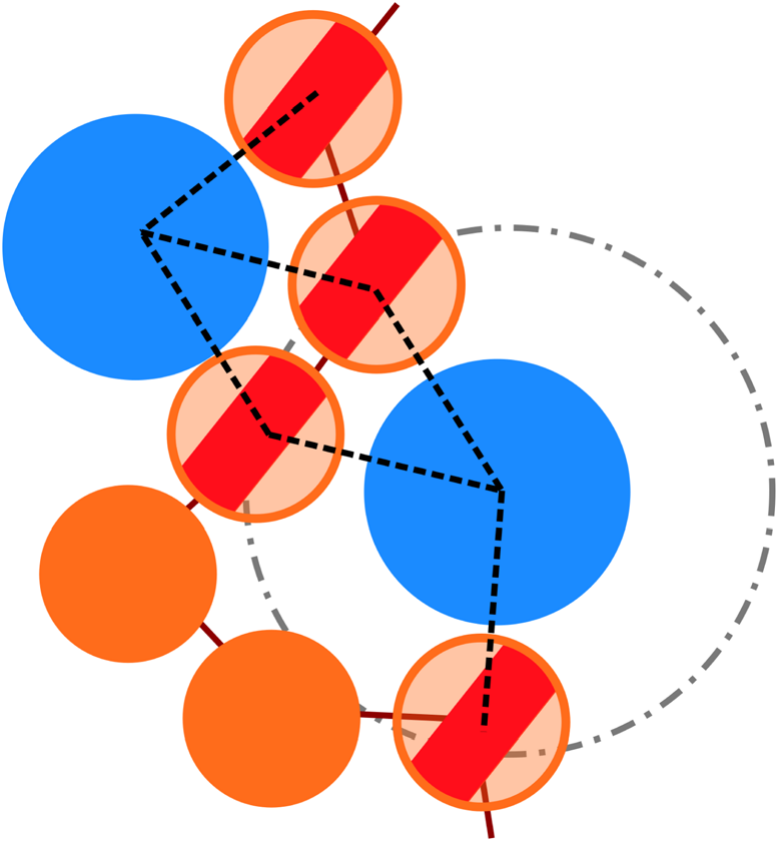
Sketch, exemplifying the difference between the number of interacting monomers *M*_int_ and the number of bonds *M*_bonds_ between colloids and monomers, drawn as dashed lines. The blue larger particles identify the colloids, while the orange smaller and tethered particles belong to the grafted chains. The dashed and dotted circle refers to the cut-off radius, employed in the analysis.

More quantitatively, we characterise adsorption at different colloid packing fractions *η*_c_ by means of four quantities. We consider (i) the average fraction of adsorption sites (*i.e.* interacting monomers) over the total number of monomers 〈*M*_int_〉/*M*; (ii) the average fraction of adsorbed colloids over their total number 〈*n*^a^_c_〉/*n*_c_; (iii) the average number of adsorption sites over the number of adsorbed colloids 〈*M*_int_/*n*^a^_c_〉 (iv) the average number of contacts or bonds between monomers and colloids 〈*M*_bonds_/*n*^a^_c_〉. The sketch in Fig. 4 illustrates the difference between these four quantities. In the sketch, the dashed and dotted circle represents the distance cut-off below which we have adsorption; the distance between any two particles (colloid or monomer) is, in the coarse-grained model used, always taken as the distance between their centres of mass. Both colloids (the blue circles) are adsorbed; instead, only the striped monomers are adsorbing sites in bad solvent, while the ones in solid color are in good solvent. Naively counting the contacts between monomers and colloids, highlighted by dashed lines in the sketch, overcounts the interacting monomers, due to the “sharing” of some adsorbing sites between colloids. This is indeed the difference between 〈*M*_int_/*n*^a^_c_〉 and 〈*M*_bonds_/*n*^a^_c_〉. Thus, the observed clustering requires paying special attention to the way the neighbours of the colloidal particles are defined and counted. In order to properly compute *M*_int_ and *n*^a^_c_, a suitable cut-off distance for adsorption should be identified; the most obvious choice, the cut-off distance of the LJ potential, leads to consistent overestimation of *M*_int_, as also the monomers in the second coordination shell will be counted. We employ the SANN algorithm,^30^ which allows for a parameter-free identification of neighbouring particles, to determine a cut-off distance.

**Fig. 5:**
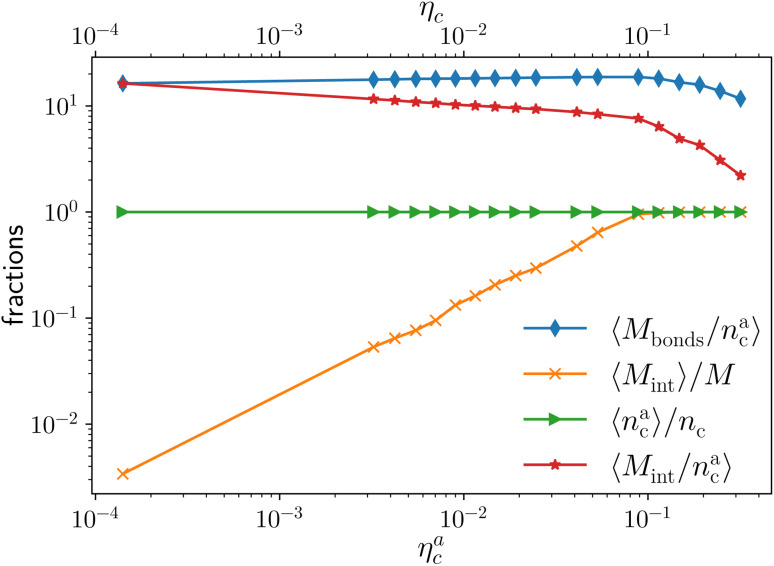
Average fraction of adsorption sites *M*_int_/*M* (orange crosses), average fraction of adsorbed colloids *n*^a^_c_/*n*_c_ (green triangles), average number of adsorption sites per adsorbed monomer *M*_int_/*n*^a^_c_ (red stars), and average number of contacts per adsorbed colloid *M*_bonds_/*n*^a^_c_ (blue diamonds) as a function of *η*^a^_c_ for the reference system *ε*_mc_ = 4*k*_B_*T*, *σ*_g_*a*^2^ = 0.064. The alternative axis, with the corresponding values of *η*_c_, is reported here for completeness, as *η*_c_ = *η*^a^_c_ for *ε*_mc_ = 4*k*_B_*T* in the range of values considered in this work.

We report the four quantities, introduced above, in Fig. 5 for the case of *ε*_mc_ = 4*k*_B_*T* and *σ*_g_*a*^2^ = 0.064. As will emerge in Section 4.3, the key quantity of interest is the average fraction of adsorbing sites per adsorbed colloid, that we name *γ*_int_ = *M*_int_/*n*^a^_c_. We can observe, in Fig. 5, that the data, reported with star-shaped symbols, present two distinct power law regimes, joined at 
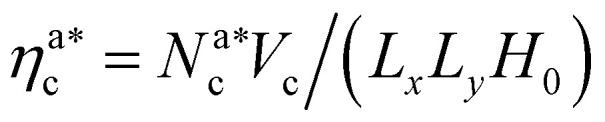
, with 
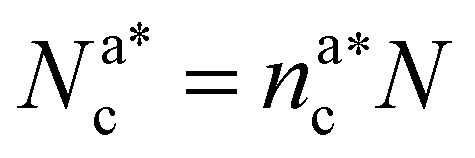
, *N* being the number of chains. 
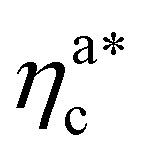
 is the value of the packing fraction at which the loaded brush collapses and there are no more non-interacting monomers. We find that, at 
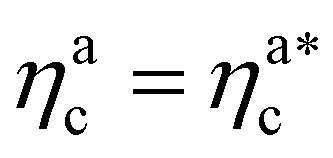
, the number of interacting monomers per adsorbed colloid is, for all analysed systems, compatible with the coordination number of a random loose packing of spheres, which is roughly 
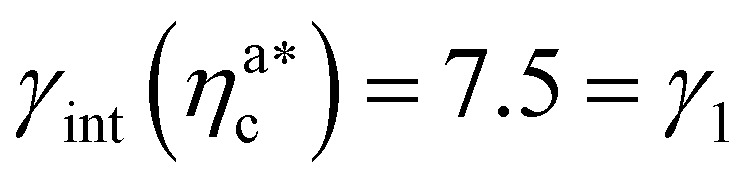
.^31,32^ The two regimes can be rationalised as follows. When very few colloids are present, each nanoparticle interacts with the maximum possible number of monomers per colloids, *γ*_int_(*η*^a^_c_ ≈ 0) = *γ*_0_ (see the ESI†); upon increasing *η*^a^_c_, but remaining below 
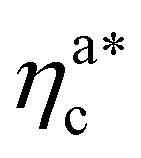
, the aggregation process imposes the colloidal particles to share some monomers, as sketched in Fig. 4. This sharing effectively lowers the ratio of interacting monomers per adsorbed colloid. Finally, above 
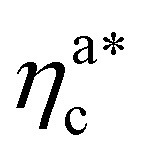
, the maximum amount of available monomers (per chain) has been reached. However, as mentioned, the brush can still adsorb additional colloids; this implies that the number of interacting sites per adsorbed colloid has to change (see Section 4.3).

The fraction of adsorbing monomers *θ* = *M*_int_/*M* is, therefore, a monotonically increasing function, that saturates to 1 at the same value of 
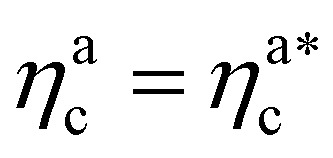
 where the collapse of the brush is observed (see Fig. 5). This implies that, above 
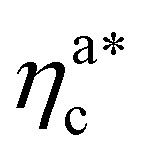
, the brush is under bad solvent conditions and, from that point on, the average height of the loaded brush will grow only due to the additional layer of colloids that deposit on the surface of the brush.

Qualitatively, the effects of adsorption on the brush, as shown in Fig. 3 and 5, can be found for *ε*_mc_ > 1*k*_B_*T* and for all the values of *σ*_g_ considered (see the ESI†). Quantitatively, the average properties of the brushes are a function of *ε*_mc_. This is due to the fact that the ability of the monomers to rearrange around the adsorbed colloids is strongly dependent on the interaction strength between monomers and colloids. In particular, for the *ε*_mc_ < 4*k*_B_*T* cases, we reach a limit in *η*^max^_c_ above which a fraction of colloids remains unadsorbed and freely diffuses in the simulation box. For *ε*_mc_ = 4*k*_B_*T* we did not reach *η*^max^_c_; thus the system has not yet reached its maximum loading capability within the range of *η*_c_ considered.

The behaviour of the system remains qualitatively similar also upon changing *σ*_g_ at fixed *ε*_mc_. However, quantitatively, varying *σ*_g_ has two main effects. First, the absolute height of the collapsed brush depends on the grafting density and is higher at higher values of *σ*_g_. Interestingly, at lower values of *σ*_g_ the brush has a lower adsorption capability. Indeed, after the collapse, brushes with a lower value of *σ*_g_ are less able to further adsorb colloids (see the ESI†). Thus, increasing *σ*_g_ pushes the adsorption limit further, similarly to an increase in binding affinity.

### 4.3 Introducing adsorbed colloids in the generalised Alexander-de Gennes scaling theory

In this section, we use the insight gained by the analysis of the numerical data to model the adsorption process in equilibrium, within the AdG scaling theory described in Section 3. As already mentioned, the key point to apply eqn (15) to a brush, that has adsorbed *n*^a^_c_ colloids per chain, is to know *M*_A_ and *M*_B_, *i.e.* the number of monomers in good and bad solvent per chain, respectively.

We recall that, within the diblock copolymer mapping introduced in Section 3, the number of monomers per chain in bad solvent *M*_B_ is given by eqn (5). We can formally rewrite eqn (5) as

16 *M*_B_ = (*γ*_int_ + *m*_B_)*n*^a^_c_

where, as described in the previous section, *n*^a^_c_*γ*_int_ = *M*_int_ accounts for the interacting monomers and *m*_B_ ∼ (*a*_c_/*a*_m_)^1/*ν*_B_^ encodes for the size difference between each adsorbed colloid and the “replacement” monomers, as introduced in eqn (5). Furthermore, *γ*_int_ introduced in Section 4.2, has a non-trivial dependence on the number of adsorbed colloids and displays two distinct regimes (see Section 4.2).

It will be convenient, from now on, to discuss the two adsorption regimes separately. The first regime deals with the properties of the brush prior to the saturation of the interacting monomers, *i.e.* when 
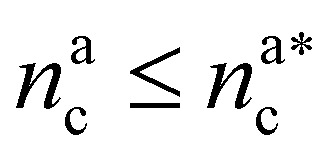
. The second regime instead refers to the properties of the brush once all interacting sites are saturated *i.e.* when 
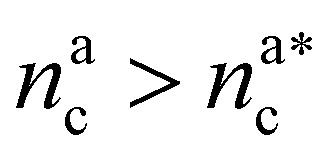
.

As noticeable in Fig. 5, data suggest that the function *γ*_int_(*n*^a^_c_) shows two different power law behaviours in the two regimes (for a more in depth discussion of the functional dependence see the ESI†).

For 
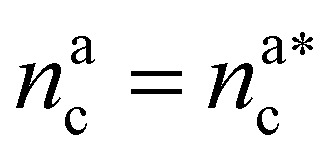
 the brush reaches its collapse. The number of adsorption sites equals the total number of monomers in the brush, *i.e. M*_int_ = *M* and *γ*_int_ = *γ*_1_.

For 
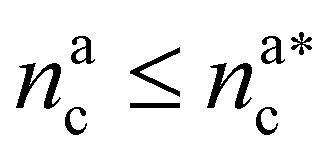
 we can define

17 *γ*_int_ = *γ*_0_(*n*^a^_c_)^−*ζ*^,

where *γ*_0_ is the number of interacting monomers in the limit of a single adsorbed colloid (see Section 4.2 and the ESI†) and *ζ* = ln(*γ*_0_/*γ*_1_)/ln(*M*/*γ*_1_). As 
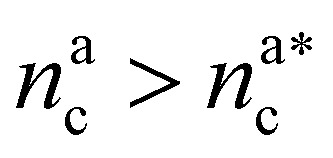
, the number of interacting monomers remains constant and is equal to *M*. As the brush still adsorbs colloids, the average number of interactions between monomers and colloids has to diminish so that:18
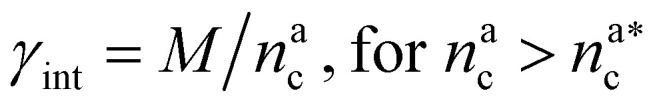


Throughout adsorption, the number of monomers in good solvent per grafted chain is given by *M*_A_ = *M* − *M*_int_, while the number of monomers in bad solvent becomes *M*_int_ + *m*_B_*n*^a^_c_. This leads to the following expression for the radius of gyration as a function of adsorption:

19 (*R*^db^_g_)^2^ = (*ξ*_B_(*M*_int_ + *m*_B_*n*^a^_c_)^*ν*_B_^)^2^ + (*ξ*_A_(*M* − *M*_int_)^*ν*_A_^)^2^

We can make the dependence of the fraction of interacting monomers *θ* on *γ*_int_ explicit, obtaining:20
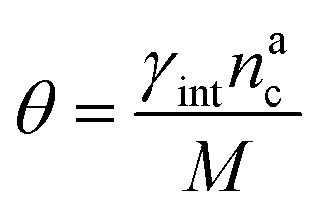
where, obviously, *θ* ∈ [0, 1]. In particular, for 
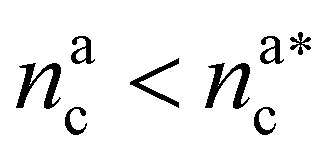
, *θ* reads21
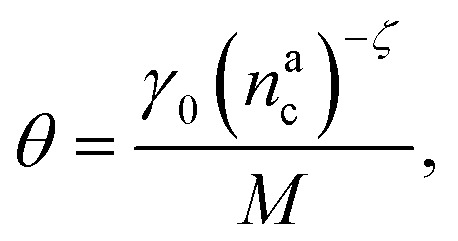
while *θ* = 1 for 
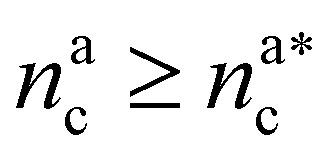
.

We can now combine eqn (11) and (21) to obtain for the case 
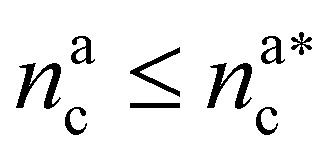
:22
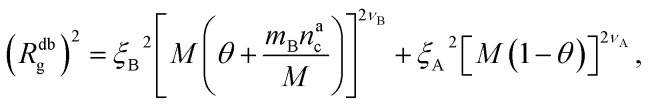
while for 
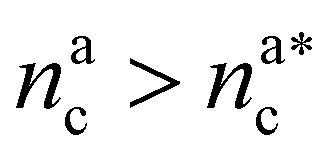
 we obtain:

23 (*R*^db^_g_)^2^ = *ξ*_B_^2^(*M* + *m*_B_*n*^a^_c_)^2*ν*_B_^.

We can now plug the results obtained for the effective diblock radius of gyration in eqn (15) to get a prediction for the height of the brush in the different regimes, obtaining, for 
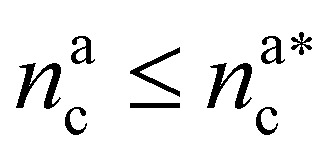
24
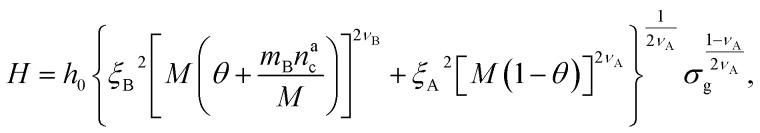
and for 
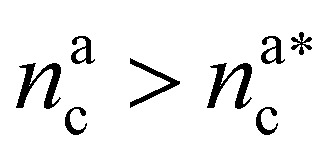
:25
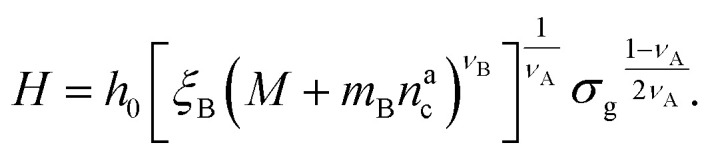


We remark that the scaling has to reproduce the free brush case for *θ* = *n*^a^_c_ = 0. This leads to a further assumption on the numerical pre-factor *h*_0_; for continuity – irrespective of the regime analysed – it has to be the same. We will then use *h*_0_ = 1.4 as done in eqn (15).

**Fig. 6:**
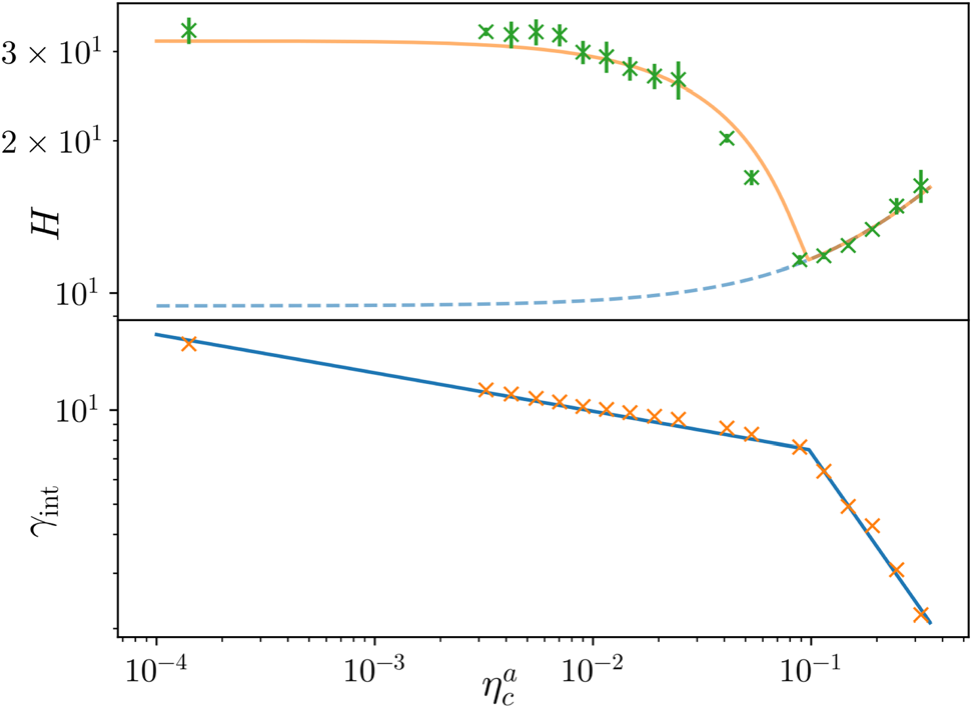
Comparison between the numerical results and theoretical predictions for both *H* (panel a) and *γ*_int_ (panel b) as a function of *η*^a^_c_ for the reference system *ε*_mc_ = 4*k*_B_*T*, *σ*_g_*a*^2^ = 0.064. In panel (a) the full line refers to the theoretical predictions of eqn (24) and (25), crosses refer to numerical data, and the dashed line refers to the fit of the data with eqn (25) (dashed line) in the second regime 
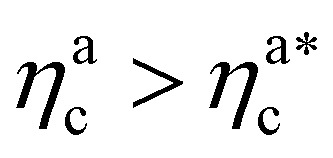
. The error bars in (a) refer to the standard deviation, determined on the five independent realisations, simulated for each value of *η*_c_.

Finally, we compare the results from the numerical simulations with those of the developed scaling theory eqn (15). We first address the case of *ε*_mc_ = 4*k*_B_*T*: we report the comparison between numerical data and theoretical prediction for the height of the brush (Fig. 6a) and for *γ*_int_ = *M*_int_/*n*^a^_c_ (Fig. 6b), as a function of *η*^a^_c_. We start from the latter panel: eqn (17) and (18) (full line) are compared with the numerical data (symbols) without any fitting parameter. The agreement is remarkable and shows that the proposed functions capture the adsorption process very well. Considering now the average height of the brush (Fig. 6a), we compare the numerical data with the predictions of eqn (24) and (25). In this case, we perform, for convenience, a fit of the data to eqn (25) (dashed line) in the second regime 
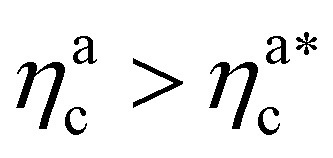
, in order to get an estimation of the prefactor *ξ*_B_ (see the ESI† for further details), assuming that it remains the same throughout the whole adsorption process. Again, the comparison between numerical data (symbols) and theoretical prediction (full line) is very favourable. Remarkably, the theory is unaware of the phase separation that is occurring at small values of *η*^a^_c_; albeit the system being heterogeneous, the picture, based on a single-chain scaling, captures the height variation very well.

We report the same comparison for the other systems considered in the ESI;† the theory always captures how the number of interacting monomers changes upon increasing the number of adsorbed colloids and yields quantitative predictions of the height of the brush, except for the case *ε*_mc_ = 1*k*_B_*T*. Indeed, in this case, the interaction affinity between monomers and colloids is not sufficient to drive the complete collapse of the brush. As the latter is one of the hypotheses of the theory, the match is not expected.

**Fig. 7:**
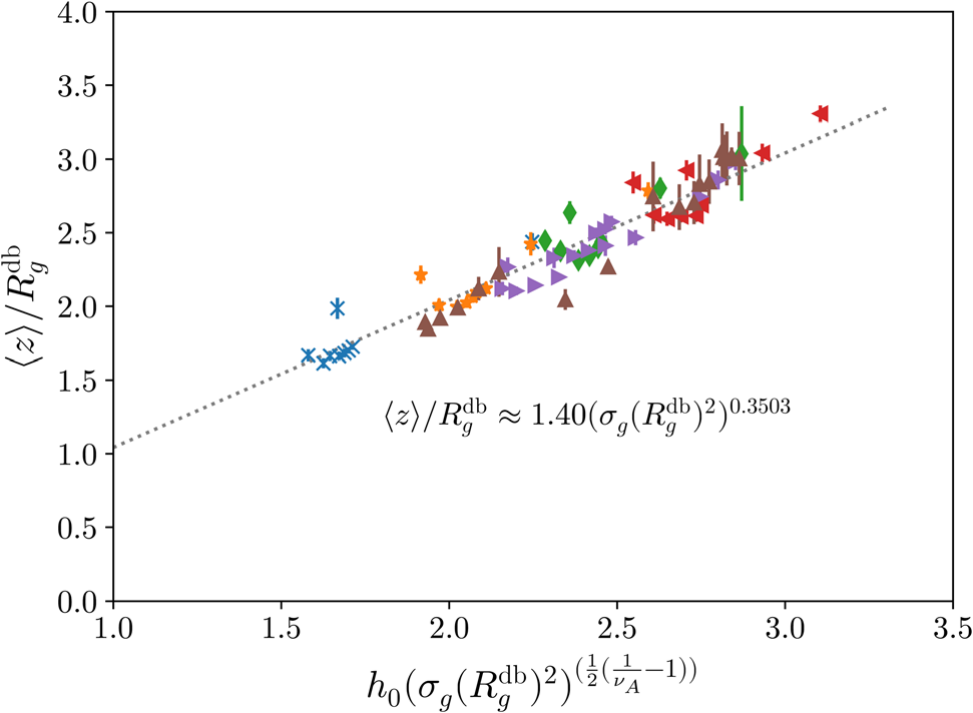
AdG scaling obtained by remapping every adsorbing brush to its own homopolymeric equivalent brush. Strikingly all systems reproduce the expected linear scaling that would be proper for the homopolymeric brush. The reported results refer to, at fixed *ε*_mc_ = 1.5*k*_B_*T*, *σ*_g_*a*^2^ = 0.032 (blue crosses), *σ*_g_*a*^2^ = 0.048 (orange stars), *σ*_g_*a*^2^ = 0.064 (green diamonds), *σ*_g_*a*^2^ = 0.08 (red left triangles) and, at fixed *σ*_g_*a*^2^ = 0.064, *ε*_mc_ = 2.0*k*_B_*T* (purple right triangles), *ε*_mc_ = 4.0*k*_B_*T* (brown up triangles).

Last, we perform a final check on our theoretical framework. Pushing the AdG theory even further, we draw a master curve, similar to that reported in Fig. 2. We report in Fig. 7 the results obtained by re-mapping all adsorbing brushes for all values of *η*_c_ and *σ*_g_ and interaction strength *ε*_mc_ between monomers and colloids, remapped onto their corresponding homopolymeric brushes and re-scaled by means of the correct AdG rescaling factor. The linear dependence of all measured 〈*z*〉/*R*_g_ values, 〈*z*〉 being the height of the brush, on the expected scaled variable 
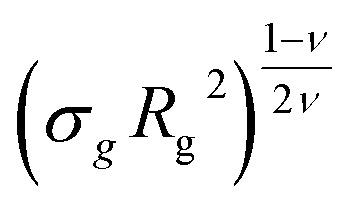
 is striking, thus corroborating and strengthening the validity of the methodology proposed here.

## 5 Conclusions

In this work we introduced a generalisation of the Alexander-de Gennes theory that allows the effects of adsorption on the average height of a brush to be quantified. The followed strategy is twofold: at a single chain level, the adsorption process is mapped onto an iterative local change in solvent quality. While interacting with colloidal particles, monomers, initially in good solvent, undergo a “phase transition”, giving rise to a diblock copolymer. The bad solvent fraction grows with the number of adsorbed colloids, that also participate in the said bad solvent fraction with a certain number of “replacement” monomers, chosen to match the colloids' occupied volume. This iterative local change in solvent quality ends when all monomers are in bad solvent. The second – and last – step in the re-mapping process consists in defining a homopolymeric brush that is “equivalent” to the newly defined diblock copolymer brush, in terms of re-scaled grafting density *σ*_g_/σ*. This continuous mapping between two descriptions of the system, the adsorbing brush and its homopolymeric “twin”, allows defining, through the AdG scaling for the “twinned” homopolymeric brush, the instantaneous value of the average height of the brush, as a function of the adsorbed colloids.

We explored systems characterised by a series of monomer–colloid interactions and diverse grafting densities. The remapping is then tested upon a wide set of simulations performed for a range of values of the monomer–colloid interaction affinity and of the grafting density *σ*_g_/σ*; it is shown to reproduce quite well the numerical results for the average height of the brush as a function of adsorbed monomers.

As mentioned, the results obtained are in agreement with those of previous theoretical approaches^14–16^ as well as with experimental observations.^28^ However, to the best of our knowledge, there are no further experimental studies that have explored this effect in detail. Rather, efforts have been focused on the applications of the brushes in other contexts.^33^

The strategy presented in this paper can thus be seen as a promising tool to monitor, *via* mesoscopic measurements, the evolution of adsorption within a homopolymeric brush through its change in average height. Such a measurement can be performed, for example, using AFM on the solvated brush;^34–36^ other techniques, such as dynamic light scattering or spectroscopic ellipsometry may be employed.^33^ Once calibrated on a specific system, universal scaling can be exploited to predict the height of the brush, given a certain concentration of colloids in the solvent. Such information is of capital importance in the design of functionalized membranes, as it allows the adsorbing capability to be maximized while, at the same time, avoiding clogging. In particular, our study suggests that, depending on the interaction affinity, an “overloaded” brush may even grow taller than in the unloaded (or unperturbed) stage. This should be avoided as, in a pore, excessive growth would lead to clogging.

We focused, in this study, on a specific size ratio between the adsorbate molecule and the adsorbing monomers; as mentioned, we chose *a*_mc_ = 1.5*a* as our case study because, typically, solvated heavy metal ions are only slightly larger than the monomers. However, we expect the results to be valid for larger size ratios within the protein limit.^37^

Finally, it could be interesting to extend a similar remapping, *i.e.* from the original system to a diblock copolymer to an equivalent homopolymer, in other settings where polymer and (in a loose sense) colloids interact to form complex structures at the nano-scale. For example, it is known that some proteins participate in the organisation of chromatin, regulating the activation and de-activation of active genes *via* a coil–globule transition that makes them more or less accessible.^38^ Furthermore, these transitions are believed to be accompanied by the condensation of the proteins,^39,40^ which was also observed in this case. As mentioned, our scaling theory, unaware of this “phase transition”, manages to reproduce the data very well. The unbalance of the protein condensation can lead to diseases, such as the Kabuki syndrome.^41,42^ Linking the protein concentration to the collapsed state of the chromatin fiber could potentially open an avenue for improving the treatment of such diseases.

## Conflicts of interest

None.

## Supplementary Material

NA-006-D3NA00598D-s001

## References

[cit1] Zhao B., Brittain W. (2000). Prog. Polym. Sci..

[cit2] Metze F. K., Klok H.-A. (2023). ACS Polym. Au.

[cit3] Barbey R., Lavanant L., Paripovic D., Schüwer N., Sugnaux C., Tugulu S., Klok H.-A. (2009). Chem. Rev..

[cit4] Feng C., Huang X. (2018). Acc. Chem. Res..

[cit5] Zhao C., Nie S., Tang M., Sun S. (2011). Prog. Polym. Sci..

[cit6] Kim S. Y., Kanamori T., Shinbo T. (2002). J. Appl. Polym. Sci..

[cit7] Keating IV J. J., Imbrogno J., Belfort G. (2016). ACS Appl. Mater. Interfaces.

[cit8] Jain P., Baker G. L., Bruening M. L. (2009). Annu. Rev. Anal. Chem..

[cit9] Sun L., Baker G. L., Bruening M. L. (2005). Macromolecules.

[cit10] He S., Zhu B., Li S., Zhang Y., Jiang X., Hon Lau C., Shao L. (2022). Sep. Purif. Technol..

[cit11] Khulbe K., Matsuura T. (2018). Appl. Water Sci..

[cit12] Vo T. S., Hossain M. M., Jeong H. M. (2020). Nano Convergence.

[cit13] Dai X., Hou C., Xu Z., Yang Y., Zhu G., Chen P., Huang Z., Yan L.-T. (2019). Entropy.

[cit14] Opferman M. G., Coalson R. D., Jasnow D., Zilman A. (2012). Phys. Rev. E: Stat., Nonlinear, Soft Matter Phys..

[cit15] Coalson R. D., Eskandari Nasrabad A., Jasnow D., Zilman A. (2015). J. Phys. Chem. B.

[cit16] Eskandari Nasrabad A., Jasnow D., Zilman A., Coalson R. D. (2016). J. Chem. Phys..

[cit17] Corsi P., De Filippo C. A., Del Galdo S., Capone B. (2022). Soft Matter.

[cit18] Kremer K., Grest G. S. (1990). J. Chem. Phys..

[cit19] Bridson R. , SIGGRAPH Sketches, 2007, 10, 1

[cit20] Thompson A. P., Aktulga H. M., Berger R., Bolintineanu D. S., Brown W. M., Crozier P. S., in’t Veld P. J., Kohlmeyer A., Moore S. G., Nguyen T. D. (2022). *et al.*. Comput. Phys. Commun..

[cit21] Alexander S. (1977). J. Phys..

[cit22] de Gennes P. G. (1980). Macromolecules.

[cit23] Binder K., Milchev A. (2012). J. Polym. Sci., Part B: Polym. Phys..

[cit24] Capone B., Likos C. N., Coluzza I. (2021). Soft Matter.

[cit25] Wittmer J., Johner A., Joanny J. F., Binder K. (1994). J. Chem. Phys..

[cit26] Coluzza I., Capone B., Hansen J.-P. (2011). Soft Matter.

[cit27] Coluzza I., Hansen J. P. (2008). Phys. Rev. Lett..

[cit28] Bano F., Carril M., Di Gianvincenzo P., Richter R. P. (2015). Langmuir.

[cit29] Ritsema van Eck G. C., Veldscholte L. B., Nijkamp J. H., de Beer S. (2020). Macromolecules.

[cit30] van Meel J. A., Filion L., Valeriani C., Frenkel D. (2012). J. Chem. Phys..

[cit31] Aste T., Saadatfar M., Senden T. (2005). Phys. Rev. E: Stat., Nonlinear, Soft Matter Phys..

[cit32] Migal L. V., Bondarev V. G., Chekanov N. A., Bondareva T. P. (2020). J. Phys.: Conf. Ser..

[cit33] Nie G., Li G., Wang L., Zhang X. (2016). Polym. Chem..

[cit34] Giessibl F. J. (2003). Rev. Mod. Phys..

[cit35] Santos N. C., Castanho M. A. (2004). Biophys. Chem..

[cit36] Dufrêne Y. F. (2008). Nat. Rev. Microbiol..

[cit37] Bolhuis P. G., Meijer E. J., Louis A. A. (2003). Phys. Rev. Lett..

[cit38] Fudenberg G., Mirny L. A. (2012). Curr. Opin. Genet. Dev..

[cit39] Erdel F., Rippe K. (2018). Biophys. J..

[cit40] Rippe K. (2022). Cold Spring Harbor Perspect. Biol..

[cit41] Banka S., Veeramachaneni R., Reardon W., Howard E., Bunstone S., Ragge N., Parker M. J., Crow Y. J., Kerr B., Kingston H. (2012). *et al.*. Eur. J. Hum. Genet..

[cit42] Fasciani A., D'Annunzio S., Poli V., Fagnocchi L., Beyes S., Michelatti D., Corazza F., Antonelli L., Gregoretti F., Oliva G. (2020). *et al.*. Nat. Genet..

