# Supplementary material for: Colloidal adsorption in planar polymeric brushes
Source: Nanoscale Adv. 2023 Oct 23;6(3):816–25. doi: 10.1039/d3na00598d (PMC10825936; doi:10.1039/d3na00598d)
Supplement: NA-006-D3NA00598D-s001 [file NA-006-D3NA00598D-s001.pdf]

Electronic Supplementary Material (ESI) for Nanoscale Advances.
This journal is © The Royal Society of Chemistry 2023

ARTICLE TYPE

Cite this: DOI: 00.0000/xxxxxxxxxx

Supplementary Informations for: Colloidal Adsorption in Planar Polymeric Brushes

Clemens Franz Vorsmann[a,b], Sara Del Galdo[c], Barbara Capone[c] and Emanuele Locatelli[a,b]

1 Bulk scaling of a single polymer

Here we briefly report the results on the simulations of homopolymer chains in the bulk. The polymer model used is the Kremer-Grest model, the same one reported in the main text. The average radii of gyrations $R_g = \xi_A M^{\nu_A}$ computed as a function of the molecular weights M are reported in semi logaritmic scale in Fig. S1 for two different solvent qualities. In panel a), we fit the model-dependent parameter ξ_A obtaining, $\xi_A = 0.487 \pm 0.006$ (fitting both the prefactor and the exponent, we get $\nu_A = 0.594 \pm 0.030$, $\xi_A = 0.474 \pm 0.064$).
Repeating the same in bad solvent in panel b) we determine $\xi_B = 0.535 \pm 0.003$ ($\nu_B = 0.302 \pm 0.001$, $\xi_B = 0.606 \pm 0.004$).

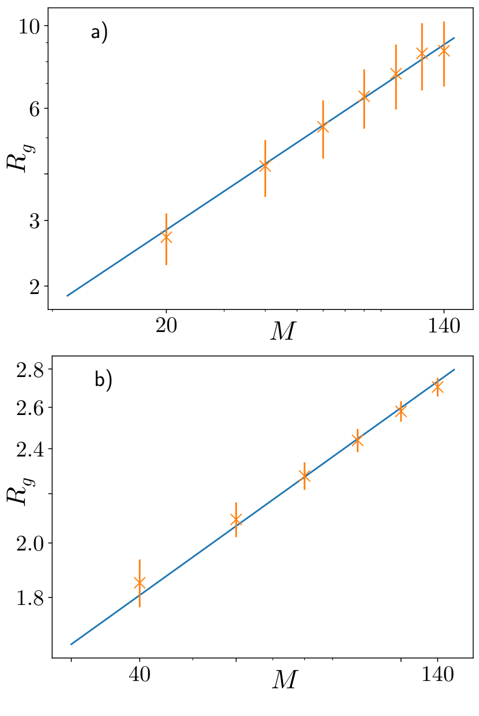

Fig. S1 Bulk gyration radius of a free polymer a) in good solvent (cutoff $\sqrt[6]{2}a$) b) in bad solvent (cutoff $2.0a$) as a function of the degree of polymerization M. Blue lines report the fit at fixed exponent ν_A =0.588, ν_B =0.33.

Dipartimento di Fisica e Astronomia, Università di Padova, via Marzolo 8, I-35131 Padova, Italy [b] INFN, Sezione di Padova, via Marzolo 8, I-35131 Padova, Italy, [c] Science Department, University of Roma Tre, Via della Vasca Navale 84, 00146, Rome, Italy

2 Scaling regime of the reference homopolymeric brush

As we will make extensive usage of Scaling Theories within our adsorption study, we report in this section on the validations of the scaling properties of the brush that has been used in this work as a reference. In particular we show, that the reference brush, composed by chains of length M =200 and grafting density $\sigma_g a^2$ =0.064, is - as expected - in the so called parabolic regime. We report, in Fig.S2 the rescaled distribution $P(z)/\sigma_g^{(1-\nu_A)/\nu_A}$ as a function of the rescaled height $z/\left(M\sigma_g^{(1-\nu_A)/(2\nu_A)}\right)$ for M =200 and several values of σ_g. The distribution $P(z)$ is, as in the main text, the probability density to find a particle at height z, measured from the bottom plane $z = 0$; $\nu_A = 0.588$ is the scaling exponent in good solvent.

Moreover, as shown in Fig. S2, we find that the profile of the brush follows the self-consistent field theory prediction[1], with a parabolic shape in the stretched regime. By rescaling the plot as the Alexander-de Gennes theory suggests, we find that all curves above $\sigma_g/\sigma^* = \sigma_g R_g^2 = 1$ fall on a master-curve, as expected, bringing further proof that the reference system is in the scaling regime.

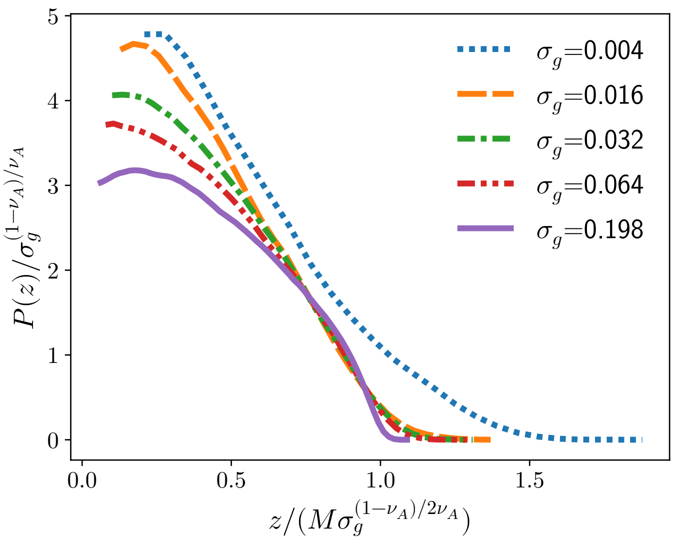

Fig. S2 Rescaled probability density $P(z)/\sigma_g^{(1-\nu_A)/\nu_A}$ as a function of the rescaled height $z/\left(M\sigma_g^{(1-\nu_A)/(2\nu_A)}\right)$ for M =200 and several values of σ_g. Values of $\sigma > \sigma_* \approx 0.009$ are expected to fall on a master-curve.

3 Equivalent diblock copolymer picture of adsorption

In this section we report some details on the mapping procedure that has been used to obtain the scaling relation for adsorbing brushes that, for the sake of clarity, we have not included extensively in the main text.

3.1 Coordination number of a single adsorbed colloid

We start from the calculation of the number of interacting monomers per adsorbed colloid in the limit of vanishing number of adsorbed colloids $\gamma_{int}(\eta_a^c \approx 0) = \gamma_0$. The calculation is approximate but it does not require input from the data.

As mentioned in the main text, we assume that, when a single colloid is adsorbed, it is surrounded by the highest possible number of monomers; we assume that, in any case, the packing fraction of the monomers around the colloid will not be different from the average packing density in the unperturbed brush. We thus consider a spherical shell around the colloid, whose radius can be quantified, in a statistical sense, as follows. Consider two particles, interacting with each other via the Lennard-Jones potential (Eq. (1) of the main text, with $a_\alpha = a_{cm}$. The average distance and its variance can be computed within the saddle-point approximation, and are given by

$$\langle r \rangle = 2^{\frac{1}{6}} a_{cm} = r_0 \tag{S1}$$

and by

$$\langle (r - r_0)^2 \rangle = \frac{a_{cm}^2}{32 \cdot 2^{\frac{2}{3}} \varepsilon_{cm}} = a_{std}^2 \tag{S2}$$

respectively. We can thus estimate the radius of the spherical shell as

$$\begin{aligned} a_{stat} &= r_0 + n_{std} a_{std} \\ &= a_{cm} \left(2^{\frac{1}{6}} + n_{std} \sqrt{\frac{1}{32 \cdot 2^{\frac{2}{3}} \varepsilon}} \right) \end{aligned} \tag{S3}$$

where $n_{std} a_{std}$ can be interpreted, assuming a Gaussian statistics, as an "interval of confidence" of the distance between the two particles. Naturally, the statistics is not really Gaussian, as the two particles cannot come close than a_{cm}, but, for our purposes, it works well. In what follows, we set n_{std} =3.

Having a suitable interaction radius, we compute $\gamma_0 = \gamma_{int}(\eta_c^a \approx 0)$ as

$$\gamma_0 = \eta_b \frac{\frac{4}{3}\pi(a_{mc} + a_{stat}/2)^3 - \frac{4}{3}\pi(a_c/2)^3}{\frac{4}{3}\pi(a_{stat}/2)^3} \tag{S4}$$

where η_b is, as mentioned, the packing fraction of the unperturbed brush

$$\eta_b = \frac{\pi}{6} \sigma_m^3 \frac{N \cdot M}{L_x L_y H_0} \tag{S5}$$

where, as in the main text, N is the number of chains, M is the number of monomers per chain, L_x, L_y are the length of the box in the x and y direction and H_0 is the height of the unperturbed brush. We can thus interpret γ_0 as the number of "effective" colloids that can be packed in the spherical shell of radius $a_{mc} + a_{stat}/2$, with packing fraction η_b.

3.2 Estimation of the local coordination number

In this section, we give some more details on the choice and on the use of the SANN algorithm.

It is not advisable to estimate the coordination number with a cut-off based method because, in most cases, the results depend on the choice of the cut-off[2]. We elected to use the SANN method to determine the number of interacting monomers around the adsorbed colloids. Briefly, the SANN algorithm identifies neighbours by "filling" the solid angle around the target particle; the distance with the farthest neighbor can be effectively taken as a cut-off distance and, accordingly, such distance may be different for each particle. Therefore, SANN can be considered as a locally adaptive algorithm[2].

We iterate the SANN algorithm over all the adsorbed colloids in the system and determine a cut-off for each of them; the resulting distributions, at different values of the colloid packing fraction η_c, are shown in S3. Interestingly, the cut-off distributions resemble a Gaussian on a first approximation, which is in agreement with the statistical approximation introduced in Section 3.1.

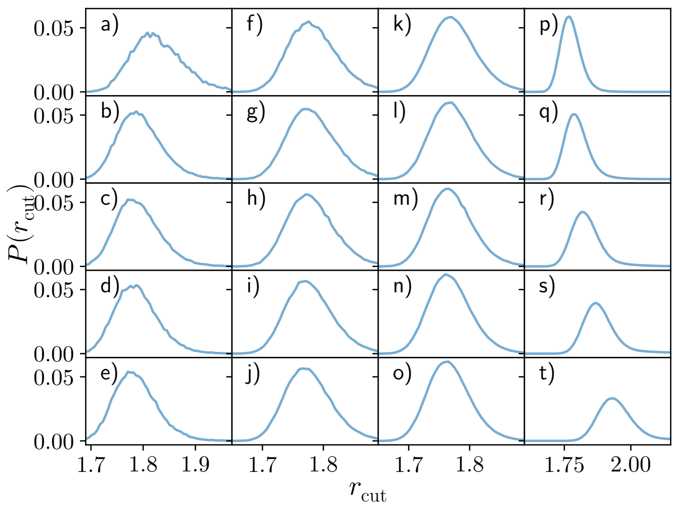

Fig. S3 Distribution of cut-off radii using the SANN algorithm for the case $\varepsilon_{mc} = 4.0 k_B T$ and $\sigma_g a_c^2 = 0.064$, reported in the main text, and several values of η_c: a) 1.30e-04 b) 3.11e-03 c) 3.74e-03 d) 4.91e-03 e) 6.51e-03 f) 8.44e-03 g) 0.01 h) 0.01 i) 0.02 j) 0.02 k) 0.03 l) 0.04 m) 0.05 n) 0.07 o) 0.09 p) 0.10 q) 0.14 r) 0.17 s) 0.23 t) 0.31.

As said in the main text, the important quantity for our mapping is the number of interacting monomers per chain M_{int}: a straightforward use of the algorithm would lead to an overestimation for the number of interacting monomers, as explained in the main text. We observed that, by itself, SANN tends to overestimate M_{int}, as it can select distant monomers as neighbours, especially if the grafting density or the colloid packing fraction is not very high. We thus determine, at fixed values of ε_{mc}, σ_g and η_c, the average cut-off distance from the distributions (e.g. Fig. S3) we find that, among the results obtained for different values of η_c, fixed ε_{mc} and σ_g, choosing the smallest value yields

the optimal results.

We remark that we preferred the SANN algorithm over something more conventional, such as fixing the cut-off as the minimum of the radial pair distribution function $g(r)$ because, in our setting, we come across two complications. First, in general, the number of adsorbed colloid is not constant in equilibrium and should be tracked. Second, we are interested in the radial distribution inside the brush and the volume of the brush is always fluctuating. Computing a reliable estimate of such a volume is not completely obvious. Thus, overall, we found the SANN approach as more suitable.

3.3 Calculation of the exponent ζ

In the main text, we report the power law exponent of the function $\gamma_{\rm int}$ as $\zeta = \ln(\gamma_0/\gamma_1)/\ln(M/\gamma_1)$. We briefly clarify here how it can be computed. Simply, if we take the logarithm of both sides of a power law such as Eq. (17) of the main text, we obtain

$$\ln(\gamma_{\rm int}) = \ln(\gamma_0) + \zeta \ln(n_{\rm c}^{\rm a}) \quad \text{(S6)}$$

thus converting the power law to a linear relation between $\ln(\gamma_{\rm int})$ and $\ln(n_{\rm c}^{\rm a})$. The exponent ζ is the slope of such a line. We can thus take the two extremes $n_{\rm c}^{\rm a} = 1$ and $n_{\rm c}^{\rm a} = n_{\rm c}^{\rm a*}$ of this first power law regime and compute the corresponding values of $\gamma_{\rm int}$, $\gamma_{\rm int}(1) = \gamma_0$ and $\gamma_{\rm int}(n_{\rm c}^{\rm a*}) = \gamma_1$. The slope is thus

$$\zeta = \frac{\ln(\gamma_1) - \ln(\gamma_0)}{\ln(n_{\rm c}^{\rm a*}) - \ln(1)} \quad \text{(S7)}$$

which gives the result in the main text upon exploiting the properties of the logarithms and substituting the definition of $n_{\rm c}^{\rm a*}$

3.4 On the fitting procedure used for Eq.(25) of the main text

As in the previous case, to simplify the fitting procedure, we take the logarithms of both sides of the equation, to switch from a power law to a linear relationship.

$$\ln H = \nu_B x^* + b \quad \text{(S8)}$$

where

$$x^* = \frac{1}{\nu_A} \ln(M + m_{\rm B} n_{\rm c}^{\rm a}) \quad \text{(S9)}$$

and

$$b = \ln(H_0 \sigma_g^{\frac{1-\nu_A}{2\nu_A}} \xi_B^{\frac{1}{\nu_{\rm B}}}) \quad \text{(S10)}$$

It is possible to fit linearly the logarithm of the data belonging to the interval $n_{\rm c}^{\rm a} > n_{\rm c}^{\rm a*}$. We perform two separate fits: in the first one, we leave both ν_B and ξ_B as fitting parameters, constraining ν_B to a lower limit $\nu_B = 0.33$. As visible in the Table S1, the values of ν_B thus obtained are compatible with the imposed lower limit. For convenience and for coherence with the picture of a collapsed brush that we have adopted so far, we fix the values of ν_B and keep only ξ_B. We report this latter fit in Fig. 6 of the main text. The estimates of ξ_B are compatible with each other; we notice that their values are much higher than the reported value of ξ_B for a bulk free chain in bad solvent (see Section 1).

4 Additional comparison between scaling theory and simulation

We report here briefly on the results obtained upon varying the binding energy $\varepsilon_{\rm mc}$ at fixed grafting density σ_g and upon varying σ_g at a fixed value of $\varepsilon_{\rm mc}$.

4.1 Comparison at fixed $\sigma_g a^2 = 0.064$

We first show, in Fig. S4, some snapshots of the system in equilibrium at fixed $\varepsilon_{\rm mc} = 2k_BT$ and different values of the colloid packing fraction $\eta_{\rm c}$.

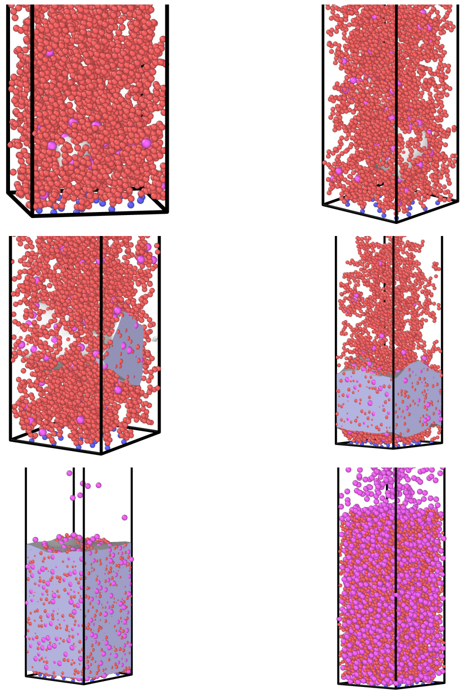

Fig. S4 Snapshots of the adsorbing brush at fixed $\varepsilon_{\rm mc} = 2.0k_BT$ and different values of $\eta_{\rm c}$ a) $\eta_{\rm c} = 0.102$, b) $\eta_{\rm c} = 0.204$, c) $\eta_{\rm c} = 0.246$, d) $\eta_{\rm c} = 0.331$, e) $\eta_{\rm c} = 0.382$, f) $\eta_{\rm c} = 0.796$.

One can immediately appreciate the same adsorption phenomenology, upon increasing $\eta_{\rm c}$, that has been described in the main text. In addition, in Fig. S4f, one can observe the "saturated" regime, where the brush is not able to adsorb any more colloids. Such regime has not been reached, within the interval of $\eta_{\rm c}$ considered, in the case reported in the main text ($\varepsilon_{\rm mc} = 4k_BT$).

In what follows, we report the same observables, discussed in the main text, for $\varepsilon_{\rm mc} = 1, 2k_BT$. We start from the probability density to find either a colloid or a monomer at height z; we report such quantity in Figs. S5,S6 where we report the case $\varepsilon_{\rm mc} = 1k_BT$ in Fig. S5 and the case $\varepsilon_{\rm mc} = 2k_BT$ in Fig. S6.

We can appreciate that, for $\varepsilon_{\rm mc} = 1k_BT$, the brush profile is not perturbed, even at high values of $\eta_{\rm c}$. Further, we can observe that, at the highest value of $\eta_{\rm c}$, there is a fraction of colloids that are not adsorbed. A different behavior emerges for $\varepsilon_{\rm mc} = 2k_BT$, where the brush deforms significantly upon adsorption and collapses, as in the main text. Nevertheless, we can observe here as well that, at the highest value of $\eta_{\rm c}$, there is a fraction of unadsorbed

ε_{mc}	σ_g	γ_0	ν_B free			ν_B fixed	
			ν_B	ξ_B	b	ξ_B	b
4.0	0.064	16.8	0.35 ± 0.03	0.9 ± 0.1	-0.9 ± 0.2	0.939 ± 0.004	-0.74 ± 0.01
2.0	0.064	15.0	0.33 ± 0.04	1.1 ± 0.3	-0.45 ± 0.39	1.11 ± 0.01	-0.45 ± 0.02
1.5	0.064	14.2	0.33 ± 0.10	1.2 ± 0.7	-0.35 ± 1.05	1.18 ± 0.01	-0.35 ± 0.02
1.0	0.064	12.9	0.33 ± 0.08	1.5 ± 0.7	0.03 ± 0.83	1.47 ± 0.02	0.03 ± 0.02

Table S1 Table of the results of the fitting of Eq. (25) of the main text in the appropriate interval $n_c^a > n_c^{a*}$.

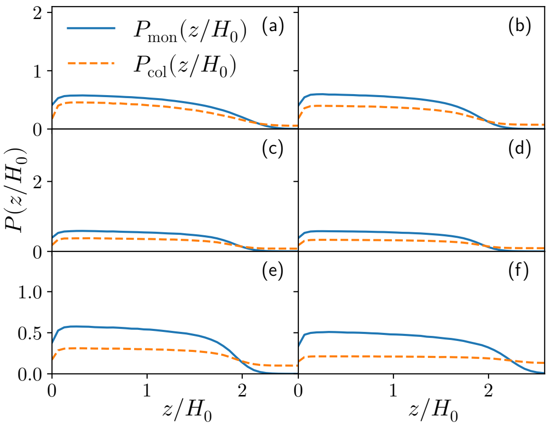

Fig. S5 Probability density $P(z)$ to find either a colloid (orange dashed line) or a monomer (blue full line) at height z as a function of the reduced height z/H_0, H_0 being the height at η_c =0, for $\varepsilon_{mc} = 1k_BT$ at different values of the colloid packing fraction a) η_c =0.102 b) η_c =0.204 c) η_c =0.246 d) η_c =0.331 e) η_c =0.382 f) η_c =0.796.

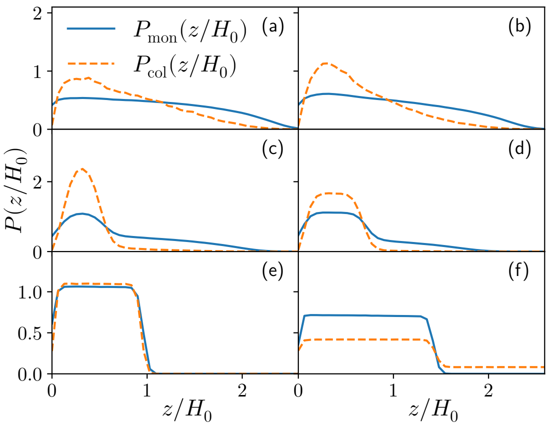

Fig. S6 Probability density $P(z)$ to find either a colloid (orange dashed line) or a monomer (blue full line) at height z as a function of the reduced height z/H_0, H_0 being the height at η_c =0, for $\varepsilon_{mc} = 2k_BT$ at different values of the colloid packing fractions a) η_c =0.00414 b) η_c =0.012 c) η_c =0.041 d) η_c =0.069 e) η_c =0.148 f) η_c =0.637

colloids, diffusing in the system.

More quantitatively, we can look at the same rates reported in the main text: i) Average fraction of adsorption sites $\theta = M_{int}/M$, ii) average fraction of adsorbed colloids n_c^a/n_c, iii) average number of adsorption sites per adsorbed monomer $\gamma_{int} = M_{int}/n_c^a$, iv) average number of contacts per adsorbed colloid M_{bonds}/n_c^a. We report the results in Fig. S7, showing the case $\varepsilon_{mc} = 1k_BT$ in panel a) and $\varepsilon_{mc} = 2k_BT$ in panel b).

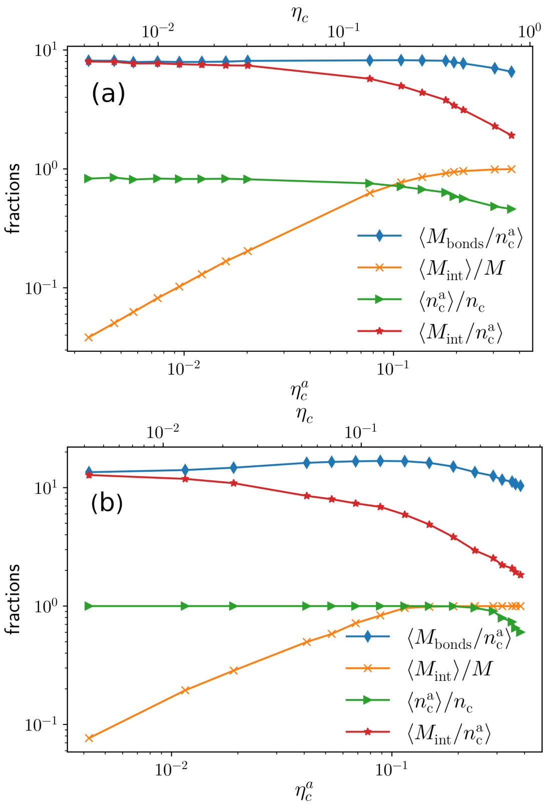

Fig. S7 Average fraction of adsorption sites M_{int}/M (orange crosses), average fraction of adsorbed colloids n_c^a/n_c (green triangles), average number of adsorption sites per adsorbed monomer M_{int}/n_c^a (red stars), average number of contacts per adsorbed colloid M_{bonds}/n_c^a (blue diamonds) as a function of η_c^a for a) $\varepsilon_{mc} = 1k_BT$, b) $\varepsilon_{mc} = 2k_BT$. The alternative axis reports the corresponding values of η_c.

By comparing the two panels between themselves and with Fig. 5 of the main panel, we can make a few interesting observations. First, the value of $\gamma_0 = \gamma_{int}(0)$ decreases upon decreasing ε_{mc}, meaning that a single adsorbed colloid is, on average, less tightly bound by the polymeric chains. This is in line with the fact that, in turn, the adsorption does not strongly modify the conformation of the chains and the profile of the brush. Interestingly, for $\varepsilon_{mc} = 1k_BT$, if η_c is high enough, the fraction of interacting monomers still reaches unity, while $n_c^a/n_c < 1$ always and start decreasing at lower values of η_c. This suggests that at $\varepsilon_{mc} = 1k_BT$ the colloids distribute evenly within the brush and, since adsorption is not strong enough, there is a dynamical equilibrium, where colloids can be exchange between the brush and the fluid. However, this is not the right scenario for most cases, where one wants

to retain the adsorbed material and, in case, release it in a controlled fashion. Instead, at $\varepsilon_{mc} = 2k_BT$, n_c^a/n_c is equal to one for a rather large interval of values of η_c and only at $\eta_c^a >> \eta_c^{a*}$ it starts decreasing, as colloids are not adsorbed anymore (see Figs. S4, S6).

Finally, we report the comparison between the scaling theory and the numerical simulations in Fig. S8 for $\varepsilon_{mc} = 1k_BT$ and in Fig. S9 for $\varepsilon_{mc} = 2k_BT$.

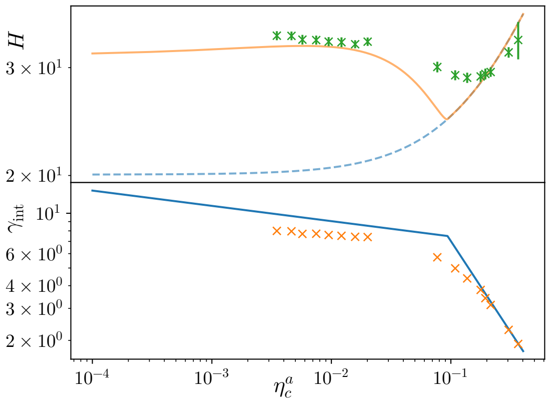

Fig. S8 Comparison between numerical results and theoretical predictions for both H and γ_{int} as a function of η_c^a for $\varepsilon_{mc} = 1k_BT$. Lines and symbols as in the main text.

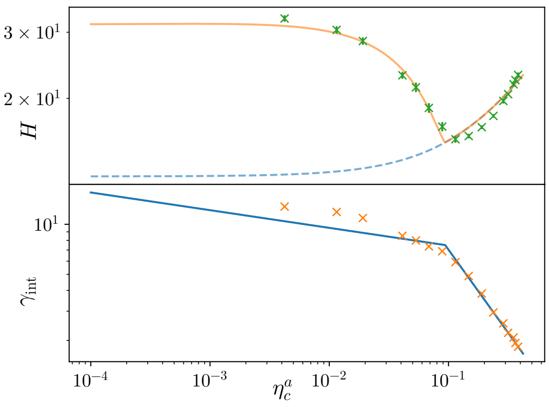

Fig. S9 Comparison between numerical results and theoretical predictions for both H and γ_{int} as a function of η_c^a for $\varepsilon_{mc} = 2k_BT$. Lines and symbols as in the main text.

While for $\varepsilon_{mc} = 2k_BT$ the theory captures both the adsorption process (through γ_{int}) and the effect of the adsorption on the brush conformation (through H), for $\varepsilon_{mc} = 1k_BT$ we only qualitatively capture the behaviour of γ_{int}. This is remarkable, and shows that the framework laid in the paper is robust. On the other hand, as mentioned in the main text, the binding affinity between monomers and colloids is not sufficient to drive the complete collapse of the brush at $\varepsilon_{mc} = 1k_BT$. As the latter is one of the hypothesis of the theory, the match can not be expected.

4.2 Comparison at fixed $\varepsilon_{mc} = 1.5k_BT$

In what follows, we report the same observables, discussed in the main text, for $\sigma_g \cdot a^2 = 0.032, 0.048, 0.064, 0.08$. We start from the probability density to find either a colloid or a monomer at height z; we report such quantity in Figs. S10-S13 where we report the different cases following the increase in $\sigma_g \cdot a^2$.

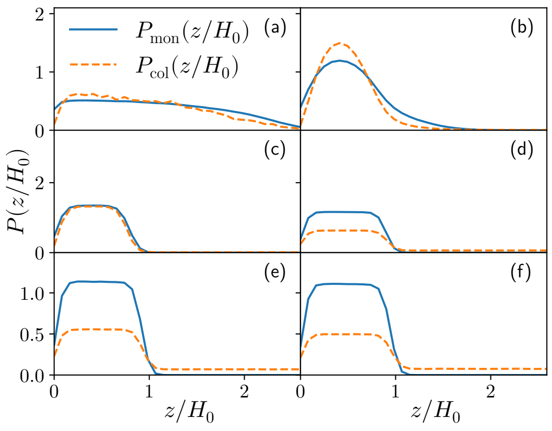

Fig. S10 Probability density $P(z)$ to find either a colloid (orange dashed line) or a monomer (blue full line) at height z as a function of the reduced height z/H_0, H_0 being the height at η_c =0, for $\sigma_g a^2 = 0.032$ at different values of the colloid packing fraction a) η_c =4.06e-04 b) η_c = 0.051 c) η_c =0.102 d) η_c =0.304 e) η_c =0.355 f) η_c =0.406

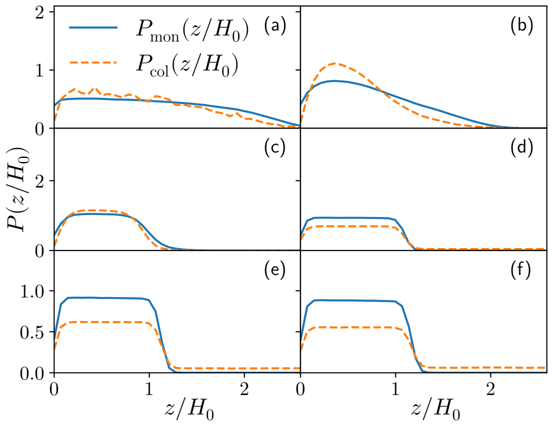

Fig. S11 Probability density $P(z)$ to find either a colloid (orange dashed line) or a monomer (blue full line) at height z as a function of the reduced height z/H_0, H_0 being height at η_c =0, for $\sigma_g a^2 = 0.048$ at different values of the colloid packing fraction a) η_c =3.52e-04 b) η_c =0.044 c) η_c =0.088 d) η_c =0.264 e) η_c =0.308 f) η_c =0.352

One can again appreciate the same adsorption phenomenology, upon increasing η_c, described in the main text and in the previous section in Figs. S10-S13. In addition, we observe that, upon increasing $\sigma_g \cdot a^2$, the brush becomes more resilient against the collapse and, in general, more a higher concentration of adsorbed colloids is needed to disturb its original parabolic profile. We can also observe that, at the highest values of η_c, the fraction of unadsorbed colloids diminishes upon increasing the grafting density. As mentioned in the main text, this makes increasing the grafting density akin to increasing the interaction energy ε_{mc}; this equivalence will be explored in the future.

More quantitatively, we can look at the same rates reported in the main text: i) Average fraction of adsorption sites $\theta = M_{int}/M$, ii) average fraction of adsorbed colloids n_c^a/n_c, iii) average num-

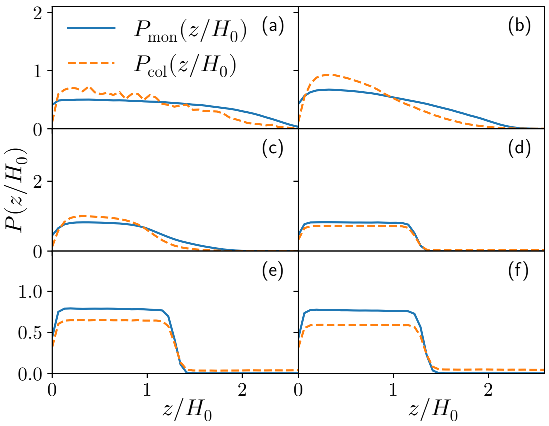

Fig. S12 Probability density $P(z)$ to find either a colloid (orange dashed line) or a monomer (blue full line) at height z as a function of the reduced height z/H_0, H_0 being the height at η_c =0, for $\sigma_g a^2$ =0.064 at different values of the colloid packing fraction a) η_c =3.18e-04 b) η_c =0.040 c) η_c =0.080 d) η_c =0.239 e) η_c =0.279 f) η_c =0.318

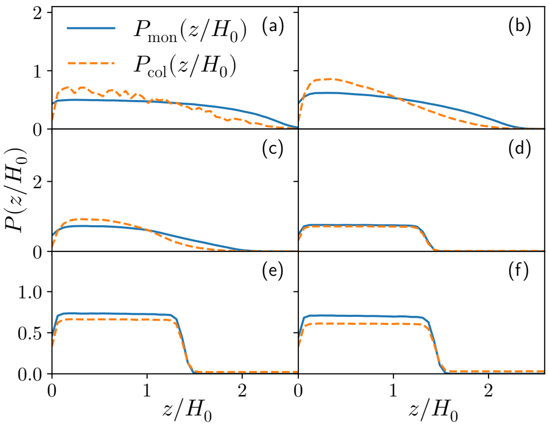

Fig. S13 Probability density $P(z)$ to find either a colloid (orange dashed line) or a monomer (blue full line) at height z as a function of the reduced height z/H_0, H_0 being the height at η_c =0, for $\sigma_g a^2 =$ 0.08 at different values of the colloid packing fraction a) η_c =2.94e-04 b) η_c =0.037 c) η_c =0.074 d) η_c =0.221 e) η_c =0.258 f) η_c =0.294

ber of adsorption sites per adsorbed monomer $\gamma_{int} = M_{int}/n_c^a$, iv) average number of contacts per adsorbed colloid M_{bonds}/n_c^a. We report the results in Fig. S14, showing the case $\sigma_g \cdot a^2$ =0.032 in panel a), $\sigma_g \cdot a^2$ =0.048 in panel b), $\sigma_g \cdot a^2$ =0.064 in panel c) and $\sigma_g \cdot a^2$ =0.08 in panel d).

By comparing the panels between themselves and with Fig. 5 of the main panel, we can make a couple of additional observations. First, the value of $\gamma_0 = \gamma_{int}(0)$ is not very sensitive on the grafting density, as it increases by a factor 1.3 upon increasing σ_g by a factor 2.5 (by roughly the same increase in ε_{mc}, the relative increase is almost a factor of 2, see Fig. S14c and Fig. 5). However it increases upon increasing σ_g, in agreement with the argument in Section 3.1: from the microscopic perspective, a denser brush will clearly have more monomers surrounding the adsorbed colloid. We also observe that the fraction of interacting monomers M_{int}/M is quite insensitive on the grafting density. Instead, as also observed before, at high values of η_c the fraction of adsorbed colloids decreases significantly more at lower values of η_c; however,

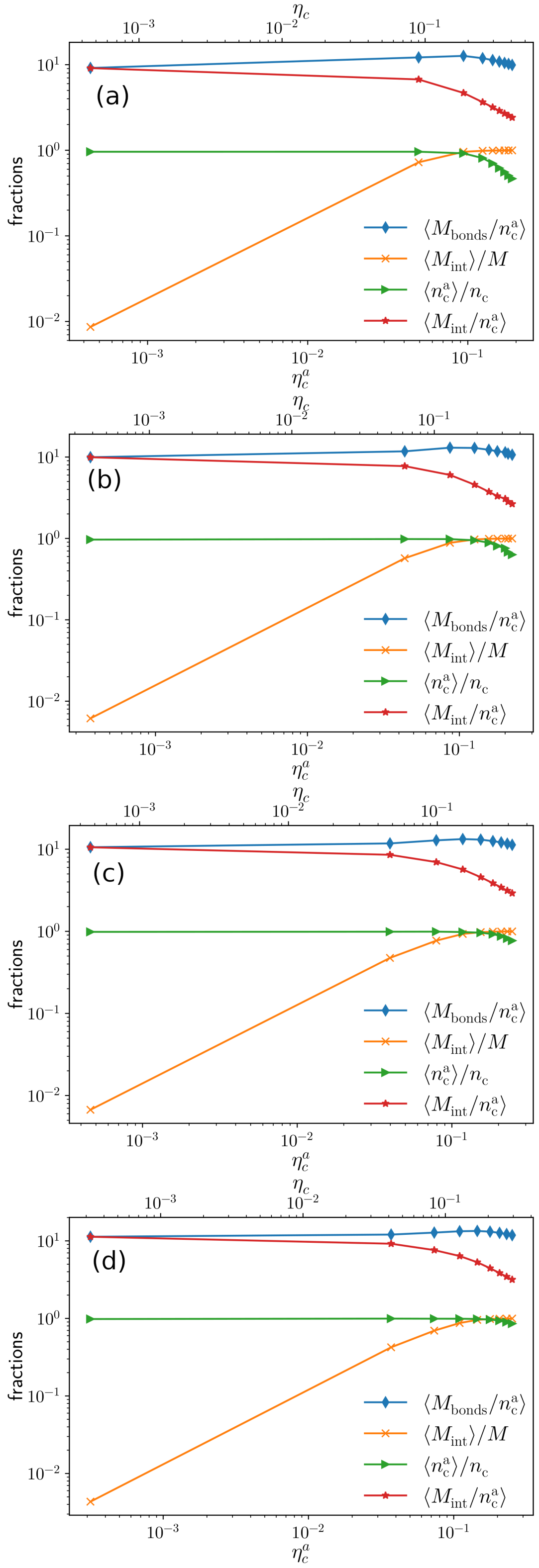

Fig. S14 Average fraction of adsorption sites M_{int}/M (orange crosses), average fraction of adsorbed colloids n_c^a/n_c (green triangles), average number of adsorption sites per adsorbed monomer M_{int}/n_c^a (red stars), average number of contacts per adsorbed colloid M_{bonds}/n_c^a (blue diamonds) as a function of η_c^a for a) $\sigma_g a^2$ =0.032, b) $\sigma_g a^2$ =0.048 c) $\sigma_g a^2$ =0.064, d) $\sigma_g a^2$ =0.08. The alternative axis reports the corresponding values of η_c.

in all cases, $n_c^a/n_c = 1$ at least below n_c^{a*}, meaning that the colloids will always be adsorbed by the brush.

Finally, we report the comparison between the scaling theory and the numerical simulations in Figs. S15-S18; again we report the different cases following the increase in $\sigma_g \cdot a^2$.

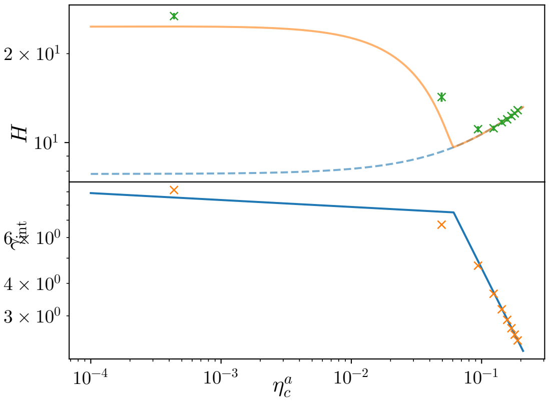

Fig. S15 Comparison between numerical results and theoretical predictions for both H and $\gamma_{\rm int}$ as a function of η_c^a for $\sigma_g a^2$ =0.032. Lines and symbols as in the main text.

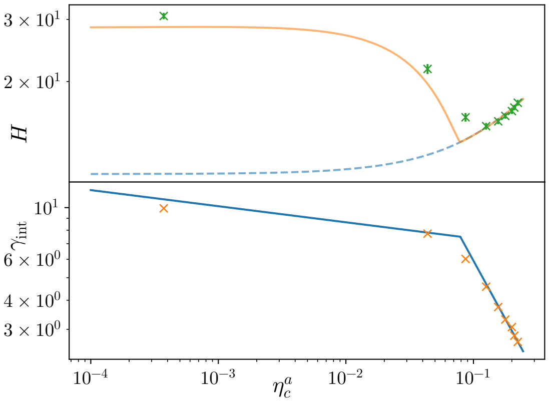

Fig. S16 Comparison between numerical results and theoretical predictions for both H and $\gamma_{\rm int}$ as a function of η_c^a for $\sigma_g a^2$ =0.048. Lines and symbols as in the main text.

The theory essentially captures both the adsorption process (through $\gamma_{\rm int}$) and the effect of the adsorption on the brush conformation (through H) for all four cases. Interestingly, the best agreement is at $\sigma_g \cdot a^2$ =0.08 and the comparison tends to be more qualitative upon lowering the grafting density. This strengthens the analogy between the rule of σ_g and $\varepsilon_{\rm mc}$ and suggest that, in order to optimize adsorption, one has to take into account both parameters.

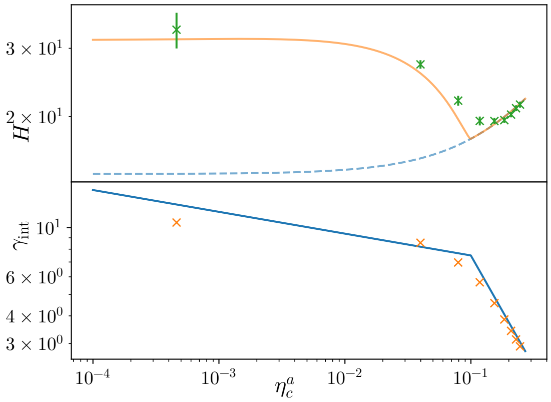

Fig. S17 Comparison between numerical results and theoretical predictions for both H and $\gamma_{\rm int}$ as a function of η_c^a for $\sigma_g a^2$ =0.064. Lines and symbols as in the main text.

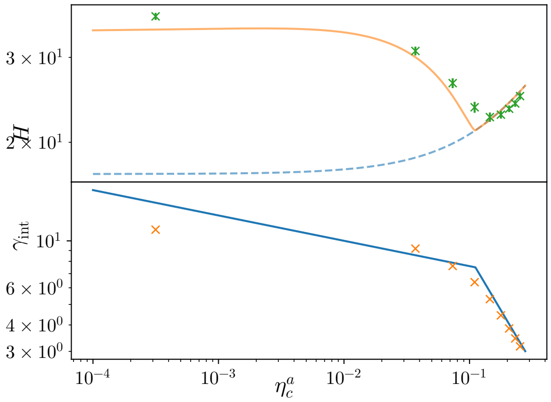

Fig. S18 Comparison between numerical results and theoretical predictions for both H and $\gamma_{\rm int}$ as a function of η_c^a for $\sigma_g a^2$ =0.08. Lines and symbols as in the main text.

Notes and references

1 Y. Zhulina, V. Pryamitsyn and O. Borisov, *Polymer Science U.S.S.R.*, 1989, **31**, 205–216.

2 R. Staub and S. N. Steinmann, *The Journal of Chemical Physics*, 2020, **152**, 024124.
